# Explaining the longitudinal interplay of personality and social relationships in the laboratory and in the field: The PILS and the CONNECT study

**DOI:** 10.1371/journal.pone.0210424

**Published:** 2019-01-30

**Authors:** Katharina Geukes, Simon M. Breil, Roos Hutteman, Steffen Nestler, Albrecht C. P. Küfner, Mitja D. Back

**Affiliations:** 1 University of Münster, Münster, Germany; 2 Utrecht University, Utrecht, Netherlands; 3 University of Leipzig, Leipzig, Germany; Sapienza University of Rome, ITALY

## Abstract

Our personalities (who we are) influence our social relationships (how we relate to people around us), and our social relationships influence our personalities. However, little is known about the specific processes underlying the complex interplay of personality and social relationships. According to the PERSOC framework, the identification of underlying social interaction processes promotes the understanding of how personality and social relationships are expressed, develop, and influence each other over time. The aim of the present paper is twofold: First, we outline and discuss four methodological challenges that arise when trying to empirically realize a process approach to the personality-relationship interplay. Second, we describe two data sets that are designed to meet these challenges and that are open for collaborative investigations: a laboratory-based process approach (Personality Interaction Laboratory Study; PILS) and a field-based process approach (CONNECT). We provide detailed information on the samples (two student samples; PILS: *N* = 311; CONNECT: *N* = 131), procedures (longitudinal and multimethodological), and measures (personality and social relationships, appearance and behavior, interpersonal perceptions), for which we present descriptive information, reliabilities, and intercorrelations. We summarize how these studies’ designs targeted the introduced methodological challenges, discuss the advantages and limitations of laboratory- and field-based process approaches, and call for their combination. We close by outlining an open research policy, aimed at accelerated collaborative efforts to further open the process black box, ultimately leading to a better understanding of the expression, development, and complex interplay of personality and social relationships.

## Introduction

**Fig 1:**
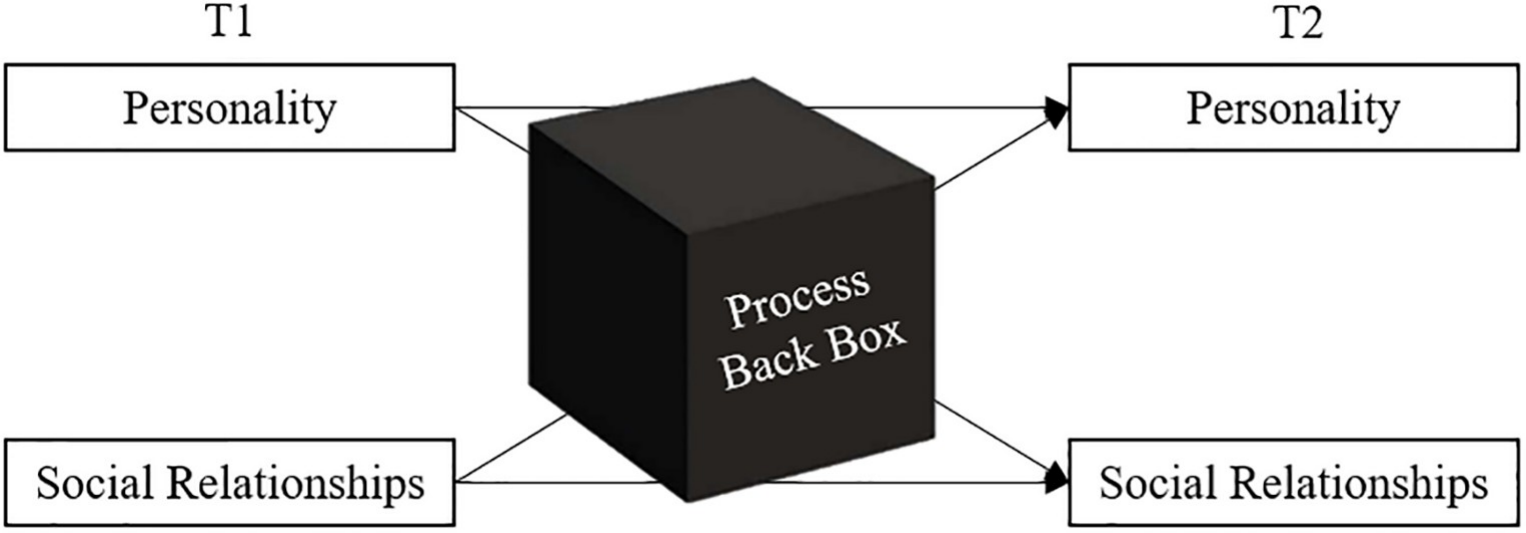
Simplified display of the process black box in the interplay of personality and social relationships over time.

Individual differences (i.e., personality) are expressed in and shape social relationships. At the same time, recurring social experiences and social representations (i.e., social relationships) characterize and feed back into people’s individuality. Despite the inextricably close link between personality and social relationships (e.g., [1–6]), researchers have traditionally focused on separately investigating either personality or social relationships. Fortunately, in the last two decades, an increasing number of researchers has started to integrate these perspectives, simultaneously investigating the mutual influence and development of personality and social relationships. This has resulted in a multitude of important insights, showing that personality influences social relationships (see [1,7] for recent overviews), and social relationships influence personality (see [8,9] for recent overviews). However, much less is known about the processes underlying these influences, the so-called process black box ([1]; Fig 1).

The goal of the present paper is twofold: It aims (1) at describing and discussing challenges that arise when trying to open this black box, and (2) at presenting two data sets designed to meet these challenges and invite the research community to use them. We will first briefly describe prior longitudinal research on personality and social relationships, identifying a need for more fine-grained, process-based, micro-level research. Second, building on a process framework for the transactional development of personality and social relationships (PERSOC; [3]), we highlight four methodological challenges that arise in the attempt to unravel personality/social relationship processes. Third, we present a laboratory- and a field-based process approach that specifically focus on these processes in the context of emerging peer relations. Fourth, we illustrate and discuss their potential to open the process black box, and call for a collaborative effort to jointly do so. Given these objectives, we would like to emphasize that this manuscript might be unconventional, unconventional in the sense that it does not aim at empirically answering open research questions. Instead, it aims at illustrating how one can realize laboratory- and field-based approaches to understand the personality-social relationship interplay and at describing two data sets that can be jointly used to do so in the context of student peer relations.

### Prior longitudinal research on personality and social relationships

Recently, a number of large-scale longitudinal studies have addressed general predictors of the development of personality traits (e.g., [10,11]) as well as social relationships [12,13]. Furthermore, an increasing number of longitudinal studies has supported the contention that personality and social relationships influence each other over time (cf., [1,8,9,14,15]). This has been most prominently shown for peer relationships, including friendships [16–21] and romantic relationships [22–25], but also for other relationship types such as family [12,26–29] and work relationships [30–32].

These studies have provided an important overview of developmental trajectories of personality and social relationships and their reciprocal influence over time. Most such studies, however, have adopted a rather macroanalytical perspective by relying on longitudinal data that span multiple years, on long time intervals, and on decontextualized assessments of potential factors of influence (e.g., retrospectively self-reported life events). To understand the processes underlying the expression, development, and reciprocal influence of personality and social relationships, a microanalytical perspective that involves fine-grained conceptualizations and assessments of process variables is needed. Findings from such an approach would considerably expand and deepen existing knowledge because they explicitly target underpinning social mechanisms and provide answers to the above mentioned process questions. But what kind of social interaction processes need to be considered?

### A process perspective on the transactional development of personality and social relationships: The PERSOC framework

PERSOC is a unifying framework for studying the dynamic interplay of personality and social relationships (PERSOC; [3]). It can be applied to all kinds of personality models (e.g., Big Five, attachment styles, interpersonal circumplex, values, and goals), relationship types (e.g., romantic, peer, and work relationships) and relationship phases (e.g., initiation and maintenance phases). The model incorporates dispositional and structural perspectives (e.g., [33]; five factor theory: [34]; theory of personality levels: [35]), conceptualizations of the transactional development of personality (identity negotiation theory: [36]; social investment theory: [37]; also see: [19,38,39]), and concepts on the nature and development of social relationships [40–44]. Importantly, the PERSOC framework combines these perspectives with a detailed analysis of relevant behavioral and perceptual processes in the social context (lens model: [45–47]; social relations model: [48,49]; models of self- and metaperceptions: [7,50]; see [51,52] and [53] for related approaches). Thus, PERSOC focuses on longitudinal social interaction processes (e.g., social behaviors and social perceptions) that take place within circumscribable social interaction units. Please note that this conceptualization of social interaction units and the included behavioral and perceptual processes has a strong resemblance to the concept of the interpersonal situation in interpersonal theory [54,55].

The basic tenet of the PERSOC framework is that both personality and social relationships are expressed in these interaction processes (i.e., in the ways in which individuals behave toward and perceive each other). Also, over time and multiple subsequent interaction units, social interaction processes are thought to mediate the development of personality and social relationships as well as their reciprocal influence. Thus, the consideration of dynamic longitudinal social interaction processes enables a comprehensive understanding of how personality and social relationships are expressed, how they develop, and how they influence each other.

### Four methodological challenges

To investigate the individual and dyadic social processes that shape the expression, development, and mutual influences of personality and social relationships, rich, longitudinal, and multimethodological data sets are necessary. Specifically, four key methodological challenges have to be addressed:

The first challenge is to capture personality development. Although personality differences are relatively stable over time, long- and short-term developments (i.e., stabilizations and changes in individual differences) are possible across the life span [20,56–59]. A successful investigation of personality stabilization and change involves an appropriate macrolevel (i.e., longer time frames) and microlevel (i.e., shorter time frames) timing of repeated trait assessments [60] (e.g., before, during, and after developmental transitions [57,61,62]). Independent of the timing, personality assessments should additionally incorporate different sources of information (i.e., self- and other perspectives) to allow for a comprehensive coverage of personality differences [63–65].

The second challenge is to capture the development of social relationships. Similar to the assessment of personality development, this challenge regards repeated assessments of long-term changes and short-term fluctuations in characteristics of social relationships. These may involve objective variables (e.g., average interaction frequency, relationship length) as well as subjective variables (e.g., interaction evaluations, relationship qualities such as relationship satisfaction, closeness, importance, and support) [3,43,66–69]. Subjective variables should capture evaluations from both individuals in a dyad to acknowledge their unique perspectives on the relationship [25]. Moreover, each individual should optimally be investigated in multiple dyads to disentangle individual (e.g., how well-accepted a person feels/is in general) and dyadic aspects of social relationships (e.g., how well a person feels/is accepted by a particular other person). Finally, to capture how social relationships develop from the beginning, the investigation should start at zero acquaintance [70–72]. Because initial phases of the acquaintance process are especially decisive for relationship development [73–77], a more fine-grained measurement of relationship indicators might capture these quick changes during the early stages of development compared to later stages.

The third challenge is to capture representative social situations. The aim is to select, create, and assess social situations that allow for valid generalizations of findings to participants’ daily life situations [45,47,78,79]. This way, typically pursued goals, typical behaviors, typical perceptions, and other typical mental states should be possible and likely to be shown or experienced. This can either be realized in standardized laboratory settings with the advantage of having a large amount of control or in real-life field settings where, by definition, representative samples of naturally occurring, truly realistic interactions can be obtained.

The fourth challenge is to capture interaction processes that take place within each interaction unit and that define the expression, development, and joint influence of personality and social relationships. According to the PERSOC framework, social interaction units are characterized by the dynamic interplay of all partners’ social behaviors and interpersonal perceptions (including self-, other-, and meta-perceptions) during the interaction. By adopting a general process perspective on personality (see [51,52] for a generic model), relevant social interaction processes can be identified as levels and contingencies of these behavioral and perceptual state expressions (level: e.g., how much a certain social behavior is expressed; how much a certain interpersonal perception is experienced; contingency: e.g., how much a certain social behavior triggers a certain interpersonal perception, and vice versa).

All of these behavioral and perceptual processes can be investigated on individual and dyadic levels. On an individual level, this includes individual differences from the actor’s perspective (i.e., actor effect; e.g., how dominant a person’s behavior toward others is in general) and the partner’s perspective (i.e., partner effect; e.g., how much dominance a person generally evokes in others), as well as their interplay (e.g., the extent to which the perception of conflict is related to behaving in a dominant way). On a dyadic (i.e., relational) level, these processes describe behavioral and perceptional state expressions and contingencies toward and from a specific social partner that cannot be explained by the described individual-level processes (i.e., relationship effect; e.g., how dominant is a person’s behavior when directed specifically toward a certain other person).

Gaining access to individual and dyadic processes involves the assessment of actual behaviors in a given social situation and the measurement of interpersonal perceptions within or closely after that social situation. Moreover, to consider all processes entirely, bidirectional behavioral and perceptual data from all persons involved in a social interaction unit are needed. This can be realized in round-robin designs (i.e., every interaction partner rates all other partners and is rated by all partners) or, when there are asymmetric groups (as in romantic encounters), in full block designs [48,49,80]. In addition, social network designs and corresponding social network analyses [81–84] are well-suited to investigate these social processes.

Despite recent developments toward more interdisciplinary and process-oriented approaches to the interplay of personality and social relationships (see [1,7] for a recent overview), longitudinal data sets that involve all relevant classes of variables to address the four abovementioned challenges are only rarely available. Such data sets are, however, sorely needed because they would enable researchers to uncover the processes by which personality and social relationships are expressed, develop, and mutually influence each other on individual and dyadic levels.

### The present approaches

We present two longitudinal multimethodological data sets, a laboratory-based approach (Personality Interaction Laboratory Study; PILS) and a field-based approach (CONNECT) on the reciprocal interplay of students’ personalities and their peer relationships in their (transition to) student life. For both data sets, we provide a chronological overview and conceptual integration of all procedures, measures, and respective data sources and present descriptive information, reliabilities, and intercorrelations of measures. We highlight their potential to answer topical process questions at the levels of the individual, the dyad, and the social network. Also, we illustrate how these approaches address the four challenges and discuss the utility of laboratory- and field based approaches and particularly of their combination. We close by outlining an open and collaborative research policy.

In PILS, we adopted a controlled laboratory-based approach that resembles the natural flow of peer interactions in the initial phase of the acquaintance process. Starting at zero acquaintance, we closely observed social interactions in small groups in three separate sessions to the point when the participants had interacted with each other for a total of approximately four hours. With its standardized and structured design, PILS focuses on direct and observable individual differences in behavior and interpersonal perceptions and their consequences for the emergence of social relationships at an early stage of acquaintance.

In CONNECT, we moved outside the laboratory and conducted a close field investigation of a newly developing peer network in its natural context, thereby also enlarging the developmental time frame. Starting at zero acquaintance with an experimental session, we followed one cohort of psychology freshmen through their whole Bachelor degree study program. CONNECT has both a short-term and long-term focus. We capture the decisive initial period of the acquaintance process with an intensively designed event- and time-based assessment and expand on these initial processes by obtaining a longer time perspective with data documenting stabilization and change in personality and social relationships within a whole peer network over almost three years.

In both studies, we assessed multiple sources of data, including individual trait measures (i.e., self- and informant-rated personality, cognitive ability measures), physical (i.e., physical attractiveness ratings) and behavioral measures (i.e., behavioral observations), interpersonal perceptions (e.g., liking, personality judgments), state measures (e.g., affect, self-esteem), as well as relationship indicators (e.g., evaluations of others’ potential to become a friend, a leader, a romantic partner, etc.). Detailed information about both studies (i.e., codebooks with comprehensive descriptions of study overviews, procedures, and measures on a global as well as single variable level) can additionally be found in the Open Science Framework (i.e., OSF; PILS: osf.io/q5zwp [85]; CONNECT: osf.io/2pmcr [86]).

In the following Method and Results sections, we describe all procedures and measures as well as descriptive results, first for the PILS and then for the CONNECT study (see Table 1; for detailed overviews of sources and domains, see S1 Table; and regarding specific variables, see S2 Table).

**Table 1:** Overview of variable groups and data sources in PILS and CONNECT.

Measure	PILS		CONNECT				
	Online survey	Session data	Zero acqu.	Online survey	Time-based	Event-based	Lab
Demographics	✔			✔			
Trait measures	✔	✔		✔			✔
Physical and behavioral measures		✔	✔			✔	✔
Interpersonal perceptions		✔	✔	✔	✔		
Relationship indicators		✔	✔	✔	✔	✔	

*Note*. Zero acqu. *=* Zero-acquaintance experiment, Lab = Laboratory-based Assessment.

## Method PILS

This Method section provides an overview of the participants, procedures, and measures used in PILS. Further details can be obtained in the codebook of this study published under osf.io/q5zwp [85].

### Participants PILS

Participants were 311 students (171 female) from various areas of study mainly recruited via email lists at the Johannes Gutenberg University in Mainz, Germany. They were between the ages of 18 and 39 (*M* = 23.80, *SD* = 3.92) and participated in exchange for research participation credit or monetary compensation (35 Euro). The sampling strategy was time-based. Prior to the start of data collection, we had decided to use a fixed time frame of 5 months and collected data from as many participants as possible within that time frame. All procedures of this study were approved by the review board of the University of Mainz and are in line with the recommendations of the DFG (German research foundation) and DGPs (German psychological society).

### Overview PILS

This study involved two phases of data collection (see Table 2).

**Table 2:** Number of participants per data source.

	*N*	Women	Men
**Online survey**			
Self-report	297	162	135
Informant- report	302	166	136
**Laboratory assessment**			
Session 1	310	170	140
Session 2	305	169	136
Session 3	298	165	133
**Summary**			
Total	311	171	140

In the first phase (henceforth referred to as the online survey), we assessed participants’ demographic information, trait measures, and additional outcome measures via self- and informant-reports in an online survey. In the second phase (i.e., laboratory assessment), we assigned the unacquainted participants to 54 small groups of 4 to 6 (in Session 1: 43 groups of 6; 9 groups of 5; 2 groups of 4), which were either same-sex or mixed-sex groups (see Table 3). They attended three sessions, taking place at a time interval of exactly 1 week.

**Table 3:** Number of groups and participants per group type and condition.

	Total (Com/Coo)	Same-sex female (Com/Coo)	Same-sex male (Com/Coo)	Mixed sex (Com/Coo)
Groups	54 (28/26)	21 (11/10)	16 (9/7)	17 (9/8)
Individuals	311 (164/147)	122 (65/57)	91 (52/39)	98 (47/51)
Women	171 (88/83)	122 (65/57)		49 (23/26)
Men	140 (76/64)		91 (52/39)	49 (24/25)

*Note*. Com = Competitive condition; Coo = Cooperative condition.

In these sessions, we investigated group interactions from zero acquaintance to short-term acquaintance with a total interaction time of approximately four hours (involving group tasks, ratings, and tests). The group interactions involved seven different tasks (Tasks A to G), three of them in Session 1 (Tasks A, B, and C), and two each in Session 2 (Tasks D and E) and Session 3 (Tasks F and G). Furthermore, in Session 2, the groups were randomly assigned to either a competitive or a cooperative condition (see Table 3).

**Fig 2:**
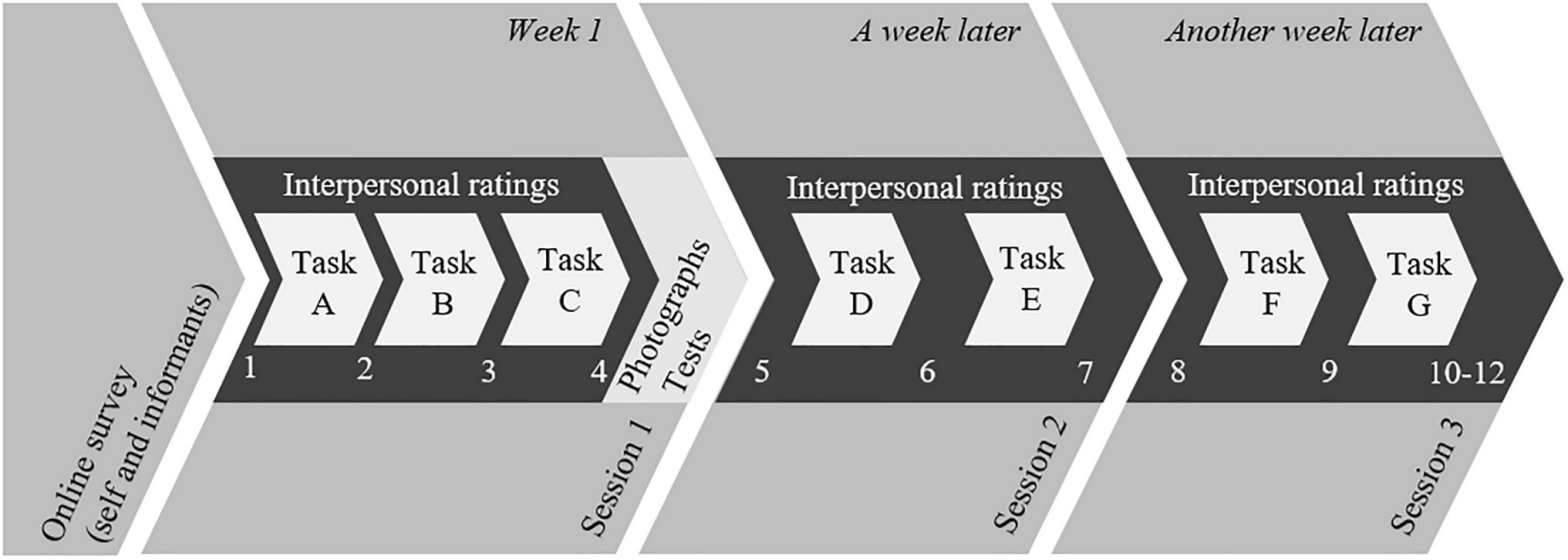
PILS’ Timeline.

At the beginning of each session and directly after each task (i.e., at 10 time points: four in Session 1; three each in Sessions 2 and 3), participants in each group rated each other regarding interpersonal perceptions and evolving relationship indicators in a round-robin design (see [48,49]) and provided their own state-affect ratings. Directly after the last rating at the end of Session 3, we obtained two further assessments: These assessments involved participants’ perceptions of dating and mating potential and of hindsight indicators referring to their memories of the zero-acquaintance rating of the interpersonal perceptions and relationship indicators at the beginning of Session 1. At the end of the first session, we additionally assessed cognitive ability measures and took two photographs of all participants. For a schematic overview, see Fig 2.

### Procedure PILS — Online survey

Students were recruited via bulletin boards, e-mail lists, and lecture announcements. Those who indicated their intention to participate in the study via email received a reply providing a link to an online survey. With this survey, we provided information about the institution’s ethical approval and assessed participants’ demographic information as well as numerous personality traits. At the end of the survey, participants were asked to enter their email addresses to receive an automatic email with a personalized link to an online survey for informant-reports that were parallelized with the self-reports. Participants were asked to send this link to (at least) two well-acquainted persons (i.e., family members or close friends) to provide us with personality informant-reports. In total, we obtained informant-reports for 302 participants. The number of informant-reports per participant ranged from 0 to 15 informants (*M* = 2.29; *SD* = 1.03; *Mdn* = 2*;* total number of informants *N* = 693; 404 female; age of informants: *M* = 25.85; *SD* = 7.83).

### Measures PILS — Online survey

#### Demographic information

The demographic questionnaire involved information about participants’ age and sex. Furthermore, we obtained data on participants’ marital status and their native languages.

#### Trait measures

By the means of questionnaires, we assessed a wide range of personality traits from the self- and informant-perspective. Wherever necessary, self-report instructions and items were rephrased to capture the informant-perspective. For descriptive results, reliabilities and correlations, please refer to the Results section.

**Big Five:** The Big Five personality dimensions were assessed with the Big Five Inventory-SOEP (BFI-S; [87]). Due to the typically low reliability of brief agreeableness scales [88,89], we added two more agreeableness items. These items were “I am a person who is generally trusting” and “I am a person who tends to find fault with others” and extended the 15-item scale to a total of 17 items. Items were answered on 7-point scales ranging from 1 (*does not apply at all*) to 7 (*applies perfectly*).

**Self-concept:** To assess participants’ self-concept, we used an extended German version of the Self-Attributes Questionnaire (SAQ; [90]). The first part of this questionnaire included the 10 original SAQ items, but the item regarding “physical attractiveness” was divided into three items referring to “physical attractiveness — face,” “physical attractiveness — body,” and “physical attractiveness — style” to mirror physical attractiveness ratings of the portrait and full-body photographs. Thus, we employed 12 items. We supplemented these original SAQ items with four items for assessing participants’ self-concepts regarding their specific intellectual abilities, in addition to the general intellectual ability as referred to in the first item. These items were intentionally chosen to correspond to those abilities assessed at the end of Session 1 (“reasoning,” “vocabulary,” “working memory,”) and “word fluency” rated on the basis of the audio tracks obtained in Task A (i.e., reading aloud, see below), respectively. In addition, we assessed participants’ self-concepts regarding their agentic (three items; “assertive,” “independent,” “ambitious”) and communal traits (four items; “helpful,” “sensitive,” “trustworthy,” “honest”). All 23 items were rated on percentile rank rating scales that provided 10 boxes to tick to indicate the belongingness to either the lower or upper 50%, 30%, 20%, 10%, or 5% of a normal distribution. Participants were asked to evaluate themselves and informants were asked to rate the participants in comparison to their same-sex peers.

**Self-esteem:** We used the 10-item German version of the Rosenberg Self-Esteem Scale (RSES; [91]) to assess self-esteem [92]. Items were answered on 4-point scales ranging from 1 (*strongly disagree*) to 4 (*strongly agree*). Aspects of self-esteem were also assessed with three additional items: “satisfied with myself,” “trusting in my abilities,” “satisfied with my appearance” (cf. [93]). These items were answered on 6-point scales ranging from 1 (*does not apply at all*) to 6 (*applies perfectly*).

**Need to belong:** We assessed participants’ need to belong with two items (cf. [94]). These items were phrased “I want others to accept me” and “I have a strong need to belong.” They were answered on 4-point scales ranging from 1 (*strongly disagree*) to 6 (*strongly agree*).

**Trait affect:** We measured selected aspects of trait affect using four single items (“active,” “optimistic,” “inhibited,” “determined”) from the Positive and Negative Affect Schedule (PANAS; [93]). These items were answered on 6-point scales ranging from 1 (*does not apply at all*) to 6 (*applies perfectly*).

**Narcissism:** To measure narcissism, we used the 40-forced-choice-item Narcissistic Personality Inventory (NPI-40; [95]; German version; [96]) and the 18-item Narcissistic Admiration and Rivalry Questionnaire (NARQ; [97]) with the subscales narcissistic admiration and rivalry. NARQ items were answered on 6-point scales ranging from 1 (*do not agree at all*) to 6 (*agree completely*).

**Dark triad:** We assessed the dark triad (narcissism, Machiavellianism, psychopathy) with the 12-item German version of the Dirty Dozen scale [98,99]. Items were answered on 9-point scales ranging from 1 (*strongly disagree*) to 9 (*strongly agree*).

**Impulsivity:** We assessed impulsivity with three selected items from the Barratt Impulsiveness Scale-11 (BIS-11; [100,101]). These items were “I tend to do things without thinking,” “I am self-controlled,” and “I act impulsively” and were answered on 6-point scales ranging from 1 (*does not apply at all*) to 6 (*applies perfectly*).

**Anger:** To measure trait anger, we employed two items from the State-Trait Anger Expression Inventory (STAXI; [102]). Selected items were “I get angry quickly” and “I am hotheaded.” They were answered on 6-point scales ranging from 1 (*does not apply at all*) to 6 (*applies perfectly*).

**Sensation seeking:** We used the German version of the Arnett Inventory Sensation Seeking (AISS-D; [103,104]) to assess participants’ tendencies to seek sensations. We selected four items: “I like to try new and exciting things,” “When I listen to music, I like it to be loud,” “I stay away from movies that are supposed to be frightening or highly suspenseful,” (reversed) and “If I were to go to an amusement park, I would prefer to ride the rollercoaster or other fast rides.” These were answered on 6-point scales ranging from 1 (*does not apply at all*) to 6 (*applies perfectly*).

**Shyness and sociability:** We assessed shyness and sociability with a total of 14 items. The first 11 items were selected and rephrased items from the shyness and sociability scales for adults [17] and answered on 5-point scales ranging from 1 (*not at all*) to 5 (*completely*). Shyness was assessed with the entire shyness subscale of five items. Sociability was assessed with three items selected from the sociability subscale (“I usually prefer to do things on my own” (reversed), “I really like to talk to other people,” “I prefer to work together with people rather than working alone”). The first three items of the shyness subscale were then employed to measure shyness toward other-sex persons. For this purpose, we replaced the word “others” with “attractive women” or “attractive men,” respectively (e.g., “I feel shy in the presence of attractive women/men,” “I feel inhibited around attractive women/men,” “I easily approach attractive women/men”). In addition, we used three selected items from the subscale “social extraversion” from the Basel State Scale (BBS; [105]). These three items with the item stems “When interacting with women, I am rather…” or “When interacting with men, I am rather…,” respectively, were answered with 5-point bipolar adjective scales (“reserved — talkative,” “distant — outgoing,” “secluded — sociable”).

**Sociosexual orientation and sexual orientation:** We assessed participants’ sociosexual orientation with the revised 9-item Socio-sexual Orientation Inventory (SOI-R; [106]). Three items with regard to the number of sexual partners (behavior subscale) were answered on a 9-point scale with its points labeled (*0*), (*1*), (*2*), (*3*), (*4*), (*5 to 6*), (*7 to 9*), (*10 to 19*), and (*20 or more*). The next three items with regard to attitudes toward sociosexuality (Attitude subscale) were answered on a 9-point scale ranging from 1 (*strongly disagree*) to 9 (*strongly agree*). The last three items pertained to frequencies of sexual fantasies and sexual arousal (Desire subscale). These items were answered on a 9-point scale with the labels (*never*), (*seldom*), (*once every 2 or 3 months*), (*about once a month*), (*about once every 2 weeks*), (*about once a week*), (*several times per week*), (*nearly every day*), and (*at least once a day*). We used the German version [107] of the Kinsey Scale (one item; [108]) to measure participants’ sexual orientation, ranging from 1 (*exclusively heterosexual*), 2 (*predominantly heterosexual*, *only incidentally homosexual*), 3 (*predominantly heterosexual but more than incidentally homosexual*), 4 (*equally heterosexual and homosexual*), 5 (*predominantly homosexual but more than incidentally heterosexual*), 6 (*predominantly homosexual*, *only incidentally heterosexual*), 7 (*exclusively homosexual*), to 8 (*no sociosexual contacts or reactions*).

#### Additional life events and outcomes

With the online survey, we obtained data on participants’ social network use. These six items involved questions about the number of hours spent on one’s favorite social network sites per week, number of friends, number of groups, number of wall posts per week, number of photo albums, and number of photos in which the subject is tagged in one’s favorite social network.

### Procedure PILS — Laboratory assessment

The scheduling of laboratory appointments was conducted in a half-automated way. Participants registered for offered time slots and, only when it was likely that already acquainted participants had registered for the same appointment (e.g., two participants studied the same subject), we rescheduled one of the respective registrations via a phone call. Prior to the three sessions of the laboratory-based assessment, participants received a standardized email and a phone call as reminders. On average, participants’ first session took place 24 days after they had completed the online survey (*M* = 23.74; *SD* = 26.52).

**Fig 3:**
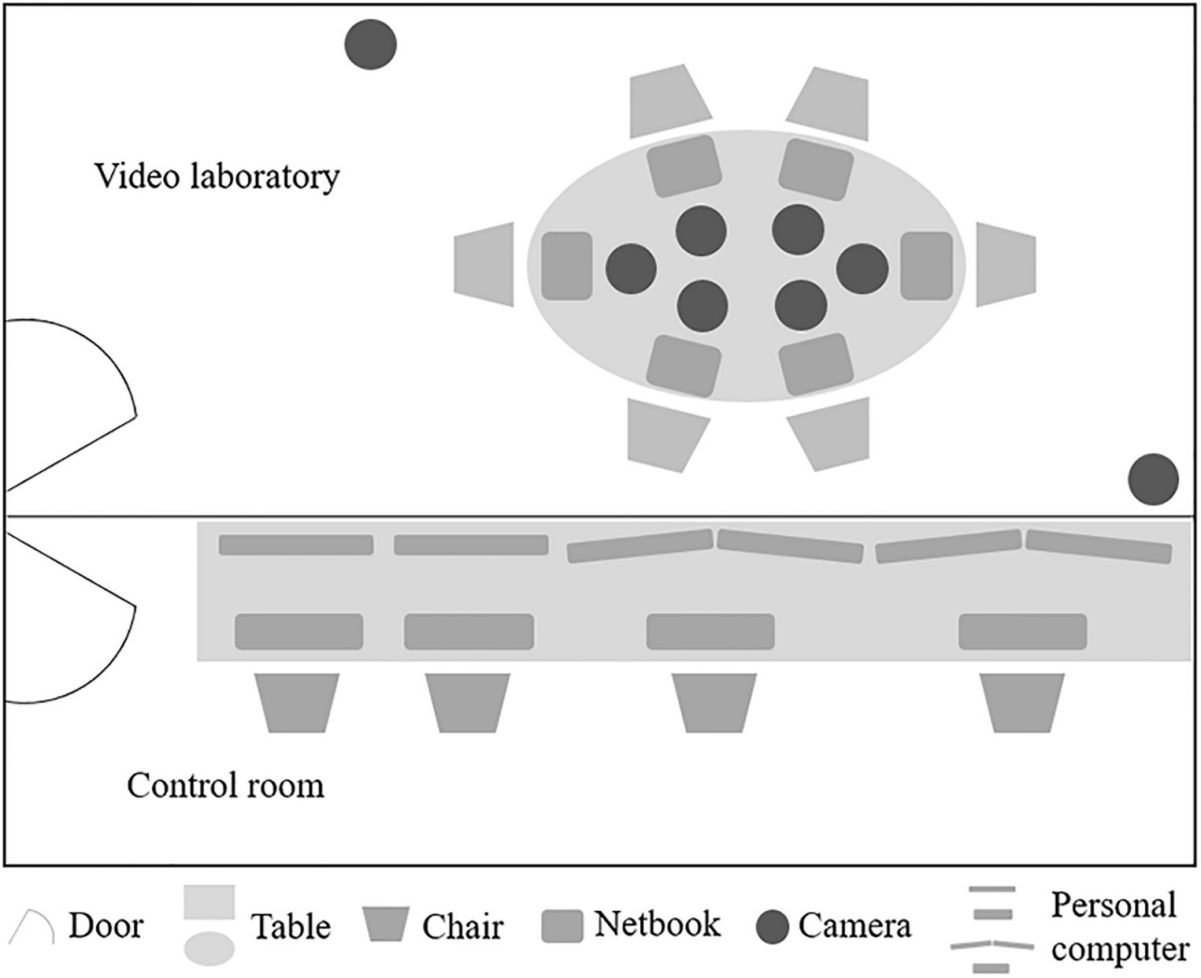
Schematic overview of the video laboratory and control room in PILS.

Setup of the video laboratory: The three sessions took place in a video laboratory (see Fig 3). In the center of the laboratory was an oval table with equally distanced and visibly numbered seating positions (i.e., clockwise). On the table, directly in front of each seating position, were netbook devices (Asus EeePC T101MT; with a pen and touch screen) for the assessment of relationship indicators, interpersonal perceptions, and state affect during the sessions.

The laboratory was equipped with a total of eight cameras. Attached to the ceiling and at opposite ends of the room were two dome cameras (resolution of 752 x 582 pixels) that recorded the groups’ interactions with a medium long shot perspective (i.e., whole group and full body). Six additional individual cameras (resolution 628 x 582) were positioned in the middle of the table for recording each participant from a close-up perspective (i.e., upper body and face) during the group interactions. A total of eight microphones recorded the sounds of the interactions. Two microphones for the recording of the overall sound were part of the dome cameras, and the other six were individually wired microphones (mini MKE2) attached to participants’ collars by a clip. Thus, a total of eight video and eight audio tracks were recorded during the group interactions. An experimenter monitored the video and sound systems from a control room next to the laboratory. The experimenter used an intercom system to provide the group with instructions and support when needed.

#### Session 1

To ensure that participants did not know each other prior to the first assessment (i.e., zero acquaintance), the experimenter and several posters outside the building and on the walls of staircases, elevators, and hallways on the way to the laboratory informed group members not to speak with each other until the experimenter indicated otherwise. The experimenter guided the participants into the video laboratory and asked them to take their designated seats. These were randomly assigned to the group members in the same-sex groups. In the mixed-sex groups, however, men and women were seated around the table in a random but alternating order. Subsequently, the experimenter welcomed the participants and gave a brief outline of the session ahead. After participants had familiarized themselves with the netbook, attached the microphones to their collars, and signed an informed consent form that also contained the institution’s ethical approval, the experimenter left the laboratory for the control room and switched on the cameras. This preparation procedure (i.e., except for ensuring zero acquaintance and signing the informed consent form) was identical in all three sessions. Before they spoke a single word to each other, participants completed the first relationship-indicator and interpersonal-perception ratings in a round-robin design. In this design, each relationship-indicator and interpersonal-perception item was first rated for oneself and then for all other group members in the clockwise order of seating positions. Subsequently, participants reported their state affect.

Participants first completed Task A (see Table 4), a reading-aloud task. For this task, participants were asked to read aloud a composition of texts, consisting of a baroque poem, a weather forecast, a sports commentary, a description of a fitness exercise, a short newspaper article, a part of a cooking recipe, and the numbers from 1 to 10 (see [109], for a related procedure). Each participant received one of six different versions of these texts. The order in which participants read these texts aloud corresponded with their seat numbers. The reading aloud task was followed by the second round-robin and state-affect ratings. Task B involved a brief self-presentation. Participants were instructed to briefly introduce themselves to the group (“Please introduce yourselves to the group, one after the other. Just say briefly who you are.”). The brief self-introduction was then followed by the third round-robin and state-affect ratings. For Task C, which involved a detailed self-presentation, participants were given more time to introduce themselves to the other group members. (“Now please introduce yourselves in more detail. Tell the others something about yourselves, about your leisure time activities and personal interests.”). The detailed self-introduction was followed by the fourth round-robin and state-affect ratings. After all participants had completed the last rating of Session 1, the experimenter switched off the cameras.

**Table 4:** Task overview for PILS.

Situation	Task description	Duration
Task A: Reading aloud	Participants read different sentences aloud, and at the end, the numbers from 1 to 10.	~ 8 min
Task B: Brief self-introduction	Participants introduced themselves briefly.	~ 2 min
Task C: Detailed self-introduction	Participants introduced themselves in a more detailed way. They talk about their interests and hobbies.	~ 6 min
**Cognitive ability & physical appearance**	Participants completed three cognitive ability tests and subsequently, the experimenter took a full body and a portrait photograph, respectively, of each participant.	~ 30 min
Task D: Lost on the moon	Participants were asked to imagine they were on a team in a space race. Because of a harsh landing, their shuttle was damaged and they had to choose from 15 objects to get to the mother ship. Their task was to decide which 12 items to choose and to rank them by importance. Cooperative condition: To find an optimal solution for the group. Competitive condition: To find an optimal solution for themselves.	~ 22 min
Task E: Ticking bomb	Participants had to discuss whether a suspected terrorist should be tortured to get information about his plan. Cooperative condition: To present a consensual group statement. Competitive condition: To present an individual statement.	~ 10 min
Task F: Moral dilemma	Participants heard a morally ambiguous, fictitious story about Marianne and Reinhard, a married couple, and three other protagonists. The groups’ task was to discuss the protagonists’ behaviors and then to rank the protagonists by their morality.	~ 13 min
Task G: Personality game	Participants first rated and explained two attributes of their own personality. Second, the whole group received a box with positive and negative personality attributes. Participants had to equally assign all attributes to all group members (two attributes each).	~ 18 min

Subsequently, all group members completed three cognitive ability tests presented on the netbooks. After participants completed the cognitive ability tests, the experimenter took one participant after another into another laboratory to take a full-body (upright format) and a portrait photograph (landscape format) of each of them. Pictures were taken in front of a white background, under standardized lighting conditions, and with identical camera settings. For both pictures, participants were instructed to look straight into the camera with a neutral (neither positive nor negative) facial expression. Hereafter, the experimenter thanked the participants for their participation in the first session and reminded them of the second session the following week.

#### Session 2

Session 2 began with the fifth round-robin and state-affect ratings and involved Task D: “Lost on the moon” (e.g., [110]) and Task E: “Ticking bomb” [111]. For the whole second session, the groups were randomly assigned to either a competitive or cooperative condition. In the competitive condition, participants were instructed that their goal was to achieve optimal individual solutions, whereas in the cooperative condition, the general goal of the discussion was to find an optimal solution for the whole group. The tasks and instructions mirrored these different goals by referring to either the participants’ individual success (e.g., “Each one of you…,” “…for your individual success,” “Your task is now…”) or to their group’s success (e.g., “You are all group members…,” “…for your group’s success,” “Your group’s task is now…”). The experimenter first provided the groups with the respective instructions for the “Lost on the moon” task (Task D). The story involved being candidates in a space race and began when they had experienced a rough landing on the moon. The candidates were 200 miles from the mother ship, which was the final destination. Most of their equipment had been damaged by the crash, and only 15 items could be used to facilitate the trip to the mothership. In both conditions, participants initially individually selected and ranked 12 of the 15 items (5 min). In the competitive condition, participants then discussed the group members’ solutions (5 min) and traded items with the other group members to attain the best selection of items for themselves (5 min). In the cooperative condition, participants selected and discussed 12 of the 15 items for a group solution (10 min). Afterwards, either each participant (competitive condition) or the group (cooperative condition) presented their results. These presentations were followed by the sixth round-robin and state-affect ratings.

Subsequently, the experimenter provided the group with the instructions for “The ticking bomb” task (i.e., Task E; [111]). The story involved the hypothetical scenario in which the lives of many people and hundreds of innocent families in the city where the study took place were in danger because a potential perpetrator was planning an imminent terrorist attack: A bomb was ticking. The perpetrator was, however, in the hands of authorities and would disclose the information needed to prevent the attack only if he was tortured. In the competitive condition, each group member was to convince the others of their own opinion about whether or not the perpetrator should be tortured. In the cooperative condition, by contrast, the group was instructed to find a common answer and prepare a group statement for the question about whether or not the potential perpetrator should be tortured to save people’s lives. After 10 min of convincing the other group members or discussing a consensual solution with them, either each participant was to prepare and present a personal statement concerning the quality of his or her own position in comparison with those of the other group members, or each group presented their common solution. These statements were followed by the seventh round-robin and state-affect ratings. Finally, the experimenter switched off the cameras, went back to the video laboratory, thanked the participants, and reminded them of the final session the following week.

#### Session 3

Session 3 began with the eighth mutual and self-ratings and first involved Task F, the discussion of a moral dilemma. The experimenter read a fictitious story about Marianne and Reinhard, a married couple. They were living together with their two kids, and as the two of them had been unemployed for some time, the family of four had only little money. The story involved the fact that some weeks ago, Reinhard was diagnosed with a rare disease that had not yet been properly researched. The only medicine that could reduce his pain was not yet approved in Germany, cost a lot of money, and needed to be purchased from abroad. This plot was the starting point for Marianne and Reinhard and three additional protagonists to experience morally ambiguous situations (e.g., spending the night with another man to get money for the medicine for her husband, investing money in opening a restaurant instead of helping his brother). To increase the probability of intense discussions and large differences in participants’ opinions, the story was pretested in order to create certain levels of ambiguity and vagueness that are typical of moral discussions in real life. Specifically, during a pretesting phase, we obtained several rounds of morality ratings of each protagonist’s behavior to modify prior versions of the story until all five protagonists received similar mean-level morality ratings while still having relatively large standard deviations across individual raters (reflecting individual differences in how the morality of the protagonists was evaluated). The groups’ task was to discuss the protagonists’ behaviors one after the other and then rank the protagonists regarding their morality (about 10 min). The presentation of this ranking was followed by the ninth round-robin and state-affect ratings.

Next, the groups played a personality game (Task G). In the first step, each participant received a box with six paper slips on which adjectives were presented. The choice of adjectives was made on the basis of German word norms of the revised Interpersonal Adjective Scales [112,113] (German Version: [114,115]). The six adjectives involved three mildly positive ones (committed, friendly, sensitive) and three mildly negative ones (reluctant, impulsive, passive) and were identical for each participant. Participants were instructed to select the two adjectives that best described their own personalities and to explain why. In the second step, all participants together received a box containing 12 adjectives—the six adjectives mentioned above plus three relatively strongly positive ones (open-minded, cooperative, well-adjusted) and three relatively strongly negative ones (manipulative, arrogant, narrow-minded). Participants were asked to jointly discuss all 12 adjectives in relation to the group members’ personalities (about 10 min). The aim was to assign the two adjectives that best described each group member. The assignment, however, had two restrictions: All 12 adjectives had to be used, and each of the adjectives could be used only once. After all participants were assigned two of the 12 paper slips, they were asked to successively provide a statement explaining why they thought they were assigned these two adjectives and whether or not they agreed with the group’s decision.

These explanations were then followed by the final and 10th round-robin and state-affect ratings. Subsequently, participants completed the round-robin ratings regarding their group members’ dating and mating potential. Finally, participants reported their memory of their perceptions of relationship indicators and interpersonal judgments at zero acquaintance (hindsight indicators). The experimenter then switched off the cameras, returned to the video laboratory, and debriefed, paid, and thanked the participants for their participation.

### Measures PILS — Laboratory assessment

#### Trait measures

Reasoning: All tests for cognitive abilities were used as Inquisit-programmed [116] and netbook-presented tests. To measure reasoning (i.e., a marker of fluid intelligence), we employed a 15-item shortened version (see [61]) of Raven’s advanced progressive matrices [117]. Each item displayed a 3 x 3 matrix. In eight of the cells, except for the bottom right one, figures that differed in a logical way following one or more abstract rules were presented. Participants were asked to select one out of eight alternative figures (presented at the bottom of the page) to logically correctly complete the 3 x 3 matrix. The raw values were the number of correctly completed matrices (theoretically ranging from 0 to 15).

**Vocabulary knowledge:** We measured vocabulary knowledge (i.e., a marker of crystallized intelligence) with the well-established German multiple-choice vocabulary test B, (Mehrfachwahl-Wortschatz-Test B; MWTB; [118]), an economic (4-to-6-min) 37-item power test. Each item consisted of one existing word as well as four non-word distractors and participants’ task was to select the existing word. Participants’ raw values were the number of correctly identified existing words (theoretically ranging from 0 to 37).

**Working memory capacity:** We assessed participants’ working memory capacity with a numeric computation span task involving dual demands regarding the transformation and storage of information [119,120]. The results of this numeric computation span task were previously found to be a valid estimator of persons’ working memory capacities (cf. [119]). The dual task involved checking whether four to eight equations were true or false (i.e., indicated by a key press) and memorizing the equations’ results (i.e., the figure after the equal sign). After all equations in a trial were presented, participants were asked to recall (i.e., type in) the results in the correct order. The equations were relatively simple addition and subtraction problems involving one- and two-digit summands and only one-digit results (e.g., 1 + 3 = 4; 17—7 = 9). The number of equations that were presented before participants were asked to recall the results increased from four to eight (i.e., two trials each), adding up to a total of 10 trials with two additional practice trials in the beginning. Participants’ raw values were the number of correctly solved trials (theoretically ranging from 0 to 10).

#### Physical and behavioral measures

Trained coders evaluated the portrait and full-body photographs as well as the groups’ and individuals’ video and audio footage to obtain physical and behavioral measures. The extensive training involved three practice sessions using sample videos. After independently rating each training video, coders jointly discussed the codings to develop a shared understanding and to ensure reliable and accurate assessments (for ICCs see Results section). In the following, we describe the initial set of obtained physical and behavioral ratings. The rich video-, photo-, and audio-based materials have yet to undergo more detailed and systematic behavioral analyses including further behavioral ratings, the assessment of behavioral frequencies and durations, the coding of specific microbehaviors, the assessment of interactive behavioral chains, and so forth. These data will allow for even more fine-grained behavioral insights.

**Physical ratings:** On the basis of the portrait photographs, trained coders rated participants’ faces regarding their physical attractiveness and their hardness (as opposed to babyfaceness) as well as their hairstyles regarding their modernness and their neat/well-groomed appearance (four items). The full-body photographs were rated regarding participants’ overall physical attractiveness and body shape (two items), their clothing (i.e., neat, modern, noticeable; three items), and their general appearance (i.e., alternative, chic; two items). All attributes were rated on scales ranging from 1 (*not at all*) to 10 (*very much so*).

**Individual behavioral ratings:** Behavioral ratings were made on relevant dimensions on 6-point scales ranging from 1 (*not at all*) to 6 (*very much so*). With the aim of providing the coders with a full picture of each dimension that captured all its facets, the rating scales involved a verbal description of high scorers’ typical behavior (i.e., behavior to be rated with a 6). All behavioral ratings were obtained using the Software INTERACT [121].

The audio tracks obtained in Task A were rated by trained coders who rated participants’ cheerfulness of voice (i.e., cheerful, happy, joyous), nervousness (i.e., nervous, stuttering, thin voice), and attention (i.e., attentive, undistracted, concentrated on task). In addition, they rated verbal fluency and intelligence (cf. [109]).

For Tasks B and C, trained coders rated the individual video footage regarding the dimensions friendly behavior (i.e., is polite, kind, considerate), expressive behavior (i.e., shows positive affect, is talkative, outgoing, active), nervous behavior (i.e., is tense, inhibited, agitated), dominant behavior (i.e., shows leadership, is confident, assertive), and arrogant behavior (i.e., is pretentious, conceited, stresses his/her own performance). After watching the self-presentation once, the coders completed their ratings; however, when coders were uncertain about their ratings on one or more dimensions, they were given the opportunity to watch the video once again.

The individual video footage of Tasks D to G was rated by trained coders on five dimensions regarding cooperative behavior (i.e., is considerate, polite, supportive), expressive behavior (i.e., shows positive affect, is talkative, outgoing, active), dominant behavior (i.e., shows leadership, is confident, assertive), arrogant behavior (i.e., is pretentious, conceited, stresses his/her own performance), and aggressive behavior (i.e., is angry, annoyed, antisocial). Due to the length of the videos, all coders watched the videos twice. After the first trial, they rated participants’ behavior on three dimensions (i.e., expressive, arrogant, and cooperative) and after the second on the remaining two (i.e., dominant and aggressive).

**Group behavioral ratings:** For Tasks D to G, six trained coders coded the group interactions from the video footage of the two dome cameras. After watching the videos, they rated the groups’ interaction processes regarding their effectiveness and performance (i.e., successful problem solving), frequency of conflicts/disharmony (i.e., frequency and intensity of conflicts, arguments), and positive atmosphere (i.e., overall positivity, good humor, and positive mood).

#### Interpersonal perceptions

In the round-robin ratings, interpersonal perceptions were assessed with nine items at all 10 time points. All items were answered on 6-point scales ranging from 1 (*does not apply at all*) to 6 (*applies perfectly*) with also 7 (*person not present*).

**Interpersonal attraction:** Four items were used to assess aspects of participants’ attraction to other group members, involving liking (“I like this person”), annoyingness (“I find this person annoying”), physical attractiveness (“This person is physically attractive”), and metaperceptions of liking (“This person likes me”).

**Personality impressions:** We used five items to assess participants’ impressions of the other group members’ personalities. These items involved perceived trustworthiness (“This person is trustworthy”), assertiveness (“This person is assertive”), intelligence (“This person is intelligent”), admiration (“This person is narcissistic and wants to be admired”), and rivalry (“This person doesn’t think much of others and wants to be better than others”).

#### Relationship indicators

Because we investigated the development of social relations at an early stage of knowing each other, relationship indicators should be interpreted as representing participants’ anticipation of relationship potential rather than already existing relationships.

**Emerging social relations:** We used two items to assess the emerging relations within the groups in each of the 10 round-robin ratings. Specifically, all group members indicated for all other group members their potential of successfully fulfilling two social roles. These items involved leadership (“I can imagine this person as a good leader”) and friendship (“I can imagine this person as a good friend”). In addition, to quantify and potentially control for the level of group members’ acquaintance prior to the experiment, their knowing (“I know this person”) was also assessed in the first round of interpersonal ratings. These items were answered on 6-point scales ranging from 1 (*does not apply at all*) to 6 (*applies perfectly*). A rating of 7 (*person not present*) indicated that the respective seat position was empty due to the group having fewer than six persons.

#### Self-perceptions and state-affect ratings

For each of the round-robin judgments, participants also reported their self-perceptions. Self-perceptions involved 10 items with the identical response format as the ratings of the other group members (1 = *does not apply at all* to 6 = *applies perfectly*) regarding relationship indicators (self-perceived relationship potential; leadership: “I can imagine myself as a good leader”; friendship: “I can imagine myself as a good friend”) and interpersonal perceptions (self-liking: “I like myself,” “I find myself annoying,” “I am physically attractive”; personality self-impressions: “I am trustworthy,” “I am assertive,” “I am intelligent,” “I am narcissistic and want to be admired,” “I don’t think much of others and want to be better than others”).

At the end of each round-robin assessment and parallel to the trait assessment, we assessed state affect with four selected items involving “active,” “optimistic,” “inhibited,” and “determined” (ranging from 1 = *does not apply at all* to 6 = *applies perfectly*). With an identical response format and also parallel to the trait assessment, state self-esteem was measured with three items: “satisfied with myself,” “trusting my abilities,” and “satisfied with my appearance.” In addition, we employed the affect grid [122], a 9 x 9 coordinate system, to measure pleasure (x-axis) and arousal (y-axis). Participants were asked to report their respective positions on both dimensions using the coordinate system on the screen, resulting in pleasure and arousal scores ranging from 1 (*negative emotion/sleepiness*) to 9 (*positive emotion/alertness*) each.

#### Dating/mating and hindsight indicators

At the end of Session 3, we measured participants’ mutually perceived dating or mating potential with four items. In the mixed-sex groups, items were phrased in the first person (i.e., “I find this person physically appealing,” “I can imagine going on a date with this person,” “I can imagine having a short love affair with this person,” “I can imagine having a long-term romantic relationship with this person”) and only completed for members of the other sex. In the same-sex groups, items were rephrased into the third person (i.e., “Persons of the other sex probably find this person physically appealing,” “Persons of the other sex can probably imagine going on a date with this person/having a short love affair with this person/having a long-term romantic relationship with this person”). In both types of groups, items capturing self-perceptions were phrased “I am physically appealing,” “Persons of the other sex can imagine going on a date with me,” “Persons of the other sex can imagine having a short love affair with me,” and “Persons of the other sex can imagine having a long-term romantic relationship with me.”

Moreover, we collected an 11th round of round-robin ratings to gather information on potential hindsight bias. Different from the other 10 time points, before and after each interactive task, participants’ did not indicate their current perceptions but their memory of their perceptions at the beginning of the first session (i.e., at zero acquaintance; “Please indicate how strongly these thoughts and feelings applied to you at the beginning of the first session”/“Please remember your ratings at the beginning of the first session”). All 10 items regarding interpersonal perceptions and relationship indicators were answered.

### Results PILS

In this part, we provide information on descriptive statistics, reliabilities, and intercorrelations for all data sources assessed in PILS with exemplary variables for each data source. All descriptive and additional exemplary analyses regarding research questions on the interplay of personality and social relationships on the individual (i.e., person-level), the dyadic (i.e., relational-level), and the network level (i.e., group-level) can be found in the online supplement of this manuscript (osf.io/zj38h/). This includes corresponding data sets and R code.

### Results PILS — Online survey

Table 5 presents a summary of the trait measures assessed in PILS. All Cronbach’s alpha scores were acceptable (ranging from.61 to.88), particularly given the brief nature of the measures (cf. [123,124]). The consistencies were similar to the ones reported in the original papers (our data vs. original studies: e.g., neuroticism.76 vs..66, extraversion.81 vs..76, openness.63 vs..58, agreeableness.63 vs..44, conscientiousness.63 vs..60 [88]; Rosenberg Self-Esteem Scale.88 vs..84 to.85 [92]; Self-Attributes Questionnaire.69 vs..76 [90]; Narcissistic Personality Inventory.80 vs..80 to.86 [96]; NARQ admiration.82 vs..87, NARQ rivalry.78 vs..83 [97]; Dirty Dozen Machiavellianism.77 vs..67 to.75, Dirty Dozen Psychopathy.60 vs..57 to.76, Dirty Dozen Narcissism.77 vs..81 to.88 [99]).

**Table 5:** PILS selected traits and cognitive abilities. Descriptives.

	Self			Informants^a^		
	*M*	*SD*	α	*M*	*SD*	α
Age	23.80	3.92		25.85	7.84	
Gender	171 ♀	139 ♂		404 ♀	288 ♂	
**Big Five**						
Neuroticism	4.31	1.34	.76	3.83	1.14	.80
Extraversion	4.79	1.24	.81	5.27	1.10	.84
Openness	4.98	1.11	.63	5.12	0.91	.67
Agreeableness	4.82	0.90	.63	5.09	0.85	.77
Conscientiousness	4.78	1.04	.63	5.36	0.94	.79
**Self**-**esteem and self-concept**						
RSES	3.10	0.55	.88	3.22	0.37	.86
SAQ	6.39	0.92	.69	6.97	0.71	.69
**Narcissism and dark triad**						
NPI^b^	14.33	6.03	.80	5.46	2.69	.80
NARQ-Admiration	3.17	0.78	.82	3.87	0.54	.75
NARQ-Rivalry	2.33	0.74	.78	2.06	0.65	.87
DIDO-Machiavellianism	3.40	1.64	.77	2.66	1.25	.82
DIDO-Psychopathy	3.17	1.48	.60	2.86	1.24	.74
DIDO-Narcissism	4.26	1.70	.77	3.60	1.40	.82
**Cognitive abilities**						
WMC — solved equations	51.17	5.93	.70			
WMC — memory span	5.19	2.27	.71			
MWTB	29.74	2.78	.63			
Short Raven	8.14	3.09	.71			

*Note*. *N*s range from 297 to 311. *M* = mean, *SD* = standard deviation, α = Cronbach’s alpha. RSES = Rosenberg Self-Esteem Scale, SAQ = Self-Attributes Questionnaire, NPI = Narcissistic Personality Inventory, NARQ = Narcissistic Admiration and Rivalry Questionnaire, DIDI = Dirty Dozen, WMC = Working memory capacity, MWTB = Vocabulary Knowledge Test [Mehrfachwahl-Wortschatz-Test]. Scales: Big Five 1 to 7, RSES 1 to 4, SAQ 1 to 10, NPI 0 to 40, NARQ 1 to 6, DIDO 1 to 9, WMC — solved equations 0 to 60, WMC — memory span 0 to 8, MWTB 0 to 36, Short Raven 0 to 15.

^a^Informant-results for *M* and *SD* refer to the mean informant-report per person. For alpha results, informant-ratings were aggregated before calculation.

^b^A short version (15 items, range 0 to 15) of the NPI was used for the informant-report.

Intercorrelations between trait variables can be found in Table 6. We found medium to high (.26 to.64 [125]) correlations between self- and informant-reports (see diagonal in Table 6).

**Table 6:** PILS selected traits and cognitive abilities. Correlations.

	1	2	3	4	5	6	7	8	9	10	11	12	13	14	15	16	17
1 Neuroticism	**.*64***	**-.14**	.09	**-.12**	.03	**-.51**	**-.40**	**-.29**	**-.27**	.10	-.01	**-.12**	.07	**-.15**	-.06	.03	-.11
2 Extraversion		**.*57***	**.21**	.11	-.02	**.30**	**.26**	**.28**	**.33**	-.10	**.15**	**-.12**	**.12**	.00	.09	**-.12**	.02
3 Openness			**.*45***	.08	.03	.03	**.20**	**.14**	**.25**	-.05	.08	-.07	.07	-.11	.01	.14	.09
4 Agreeableness				**.*38***	**.12**	**.13**	**.16**	**-.15**	-.02	**-.28**	**-.18**	**-.39**	-.06	.01	.06	.03	.01
5 Conscientiousness					**.*48***	**.13**	**.29**	**.13**	-.01	-.05	-.09	**-.24**	.04	.03	-.03	**-.14**	-.02
6 RSES						**.*49***	**.50**	**.42**	**.44**	-.07	.09	-.01	.01	**.20**	.10	-.05	.11
7 SAQ							**.*29***	**.52**	**.48**	.07	**.13**	-.01	**.12**	**.14**	.05	-.01	.06
8 NPI								**.*49***	**.67**	**.34**	**.39**	**.21**	**.41**	.10	.01	-.07	**.13**
9 NARQ-Admiration									**.*30***	**.33**	**.39**	**.21**	**.46**	**.15**	.05	-.02	**.14**
10 NARQ-Rivalry										**.*28***	**.46**	**.41**	**.44**	.03	-.04	-.07	-.02
11 DIDO-Machiavellianism											**.*27***	**.47**	**.47**	.03	.08	.06	.11
12 DIDO-Psychopathy												**.*27***	**.26**	.06	.02	.08	.04
13 DIDO-Narcissism													**.*26***	.03	.00	.01	**.15**
14 WMC — solved equations														-	**.27**	.09	**.19**
15 WMC — memory span															-	**.18**	**.15**
16 MWTB																-	**.19**
17 Short Raven																	

*Note*. *N*s range from 294 to 309. Above the diagonal: intercorrelations between self-reported traits. Diagonal elements (in italics): correlations between self-reported traits and informant-reported traits. Significant correlations (*p* <.05) in bold.

Regarding the Big Five, self-informant correlations were similar to or higher than those found in a recent meta-analysis [30] (our data vs. self-family agreement/self-friend agreement; neuroticism:.64 vs..43/.33, extraversion.57 vs..48/.40, openness:.45 vs..43/.33, agreeableness:.38 vs..37/.29, conscientiousness:.48 vs..42/.38). The correlations between the Big Five traits were also similar to meta-analytical results, albeit a bit smaller (cf. [126], our data vs. meta-analysis: extraversion and openness.21 vs..31, agreeableness and conscientiousness.12 vs..31, neuroticism and agreeableness -.12 vs. -.31, neuroticism and extraversion.-14 vs. -26). For neuroticism and conscientiousness, we did not find the expected negative association (.03 vs. -.32).

### Results PILS — Laboratory sessions

Table 7 shows the descriptive results obtained in PILS laboratory sessions. Regarding self-perceptions, we computed multilevel models (using the R package lme4: [127]) to assess the variance between perceivers and within perceivers across the 10 time points. Here, the between variance was consistently found to be higher than the within variance. That is, for example, there was more variation between participants in how likable they perceived themselves to be than within participants across the 10 time points. For other perceptions, we applied social relations model analyses (using the R package TripleR: [128]) and estimated actor variance (i.e., How much do people differ in how they generally see others?), target variance (i.e., How much do people differ in how others generally perceive them?), and relationship variance (i.e., How much do people differ with respect to their unique perceptions of specific other persons? cf. [48]). As previous research has suggested (cf. [49]), liking was for the most part a dyadic phenomenon (relationship variance: 49% of the total variance), whereas metaliking was mainly influenced by perceivers’ expectations (perceiver variance: 52% of the total variance). Also, in line with previous findings, easily observable characteristics such as attractiveness and assertiveness showed a relatively large amount of target variance (both 27% of the total variance; cf. [129]).

**Table 7:** PILS session results (self-ratings, interpersonal perceptions).

	Self-perceptions			Other perceptions			
	*M*	*SD within*	*SD between*	*M*	*Per*. *Var*.	*Tar*. *Var*.	*Rel*. *Var*.
**Interpersonal perceptions**							
Knowing				1.33	0.13	0.03	0.47
Liking	4.90	0.56	0.81	4.25	0.55	0.10	0.62
Metaliking				3.92	0.50	0.04	0.42
Annoying	1.81	0.71	0.82	1.68	0.52	0.03	0.50
Assertiveness	4.32	0.58	0.87	3.88	0.37	0.40	0.70
Trustworthiness	5.22	0.48	0.66	4.18	0.62	0.05	0.55
Intelligence	4.74	0.42	0.73	4.44	0.50	0.08	0.44
Attractiveness	4.12	0.54	1.01	3.56	0.61	0.47	0.69
Admiration	2.14	0.62	1.05	2.16	0.67	0.18	0.58
Rivalry	1.76	0.49	0.84	1.96	0.59	0.08	0.52
**State affect**							
Pleasure (affect grid)	6.34	1.13	1.23				
Arousal (affect grid)	5.73	1.22	1.24				
Active	3.78	0.84	0.86				
Optimistic	4.23	0.67	0.91				
Inhibited	2.36	0.75	0.87				
Determined	4.07	0.63	0.91				
Satisfied with myself	4.12	0.70	0.89				
Trust my abilities	4.33	0.57	0.89				
Satisfied with my appearance	3.99	0.56	1.10				
**Relationship indicators**							
Leadership	4.13	0.58	1.02	3.67	0.41	0.42	0.89
Friendship	5.03	0.68	0.72	3.87	0.60	0.16	0.99
Physical appeal*	4.17		1.18	3.86	0.32	0.96	0.75
Date*	4.11		1.23	4.05	0.41	0.78	0.82
Short love affair*	3.66		1.44	3.59	0.37	0.85	1.09
Romantic relationship*	4.24		1.28	4.42	0.35	0.31	0.87

*Note*. *N*s range from 294 to 311. *M* = mean aggregated across individual perceiver means, *SD within* = standard deviation within perceivers, *SD between* = standard deviation between perceivers. *Per*. *Var*. = Perceiver Variance, *Tar*. *Var*. = Target Variance, *Rel*. *Var*. *=* Relationship Variance. Scales: Interpersonal Perceptions, Relationship Indicators, State Affect 1 to 6, Affect grid 1 to 10. For variance analyses, results for the 10 time points were aggregated, group sizes of three or fewer participants were excluded;

*for these items, we included only the mixed-sex groups for other-perceptions.

### Results PILS — Direct observations

Table 8 displays descriptive information on behavior, attractiveness, and group ratings. These results were based on video footage, audio tracks, or photographs of participants. Ratings were obtained from three to six independent raters with an Intraclass Correlation, ICC (2,k), that ranged from.53 to.94.

**Table 8:** PILS selected behavior, attractiveness, and group ratings.

Tasks and variables	*M*	*SD*	ICC	Tasks and variables	*M*	*SD*	ICC
**Task A (**reading aloud**,** audio tracks)				**Task F** (moral dilemma, video footage)			
Cheerfulness of voice	3.18	0.76	.79	Expressiveness	3.17	1.05	.89
Nervousness	2.81	0.61	.59	Dominance	3.02	1.11	.92
Attention	3.79	0.46	.55	Arrogance	2.47	1.06	.84
Verbal fluency	3.97	0.73	.76	Cooperativeness	2.91	0.76	.74
Intelligence	3.68	0.57	.72	Aggressiveness	2.35	1.00	.84
**Task B** (brief self-intro, video footage)				**Task G** (personality game, video footage)			
Expressiveness	2.48	0.98	.64	Expressiveness	3.10	1.04	.89
Dominance	2.81	0.98	.70	Dominance	2.79	1.00	.90
Arrogance	2.16	0.89	.66	Arrogance	2.35	0.96	.84
Friendliness	3.19	1.05	.77	Cooperativeness	3.15	0.80	.78
Nervousness	2.84	0.93	.62	Aggressiveness	1.93	0.77	.81
**Task C** (detailed self-intro, video footage)				**Attractiveness** (photographs)			
Expressiveness	3.30	1.12	.76	Attractiveness face	4.35	1.43	.81
Dominance	3.22	0.93	.70	Hardness of face	5.19	1.12	.69
Arrogance	2.36	0.87	.62	Styled hair	4.71	1.31	.74
Friendliness	3.41	0.86	.72	Neat hair style	5.82	1.36	.84
Nervousness	2.60	0.80	.53	Overall attractiveness	5.11	1.30	.82
**Task D** (lost on the moon, video footage)				Body shape (*recoded)*	6.34	1.15	.80
Expressiveness	3.16	1.13	.90	Neat clothing	5.85	0.80	.60
Dominance	2.87	1.13	.91	Modern clothing	4.81	1.17	.77
Arrogance	2.22	0.99	.84	Flashy clothing	4.47	1.51	.86
Cooperativeness	2.79	0.74	.71	Alternative appearance	3.93	1.50	.80
Aggressiveness	1.92	0.85	.83	Chic appearance	4.28	1.55	.85
**Task E** (ticking bomb, video footage)				**Group ratings** (video, aggregated over tasks)			
Expressiveness	3.24	1.19	.91	Performance	3.79	1.32	.84
Dominance	3.01	1.19	.94	Frequency of conflicts	3.23	1.25	.90
Arrogance	2.30	1.03	.84	Atmosphere	3.32	1.30	.85
Cooperativeness	2.74	0.85	.78				
Aggressiveness	2.12	0.98	.86				

*Note*. *N*s range from 278 to 305 (54 groups). *M =* mean rating of participants. *SD* = standard deviation between participants/groups. ICC = intraclass correlation (2,k). Scales: Audio tracks & Video footage 1 to 6, photographs 1 to 10. All variables were rated by six (three for Tasks B and C) independent raters.

To investigate the relationship between personality and behavior shown in the laboratory, we correlated averaged behavior ratings across the 10 time points with corresponding self- and informant-reported traits, respectively (e.g., self-reported/informant-reported: nervousness and trait neuroticism.19/.11, dominance-expressiveness and trait extraversion.37/.40, friendliness-cooperativeness and trait agreeableness.11/.02, arrogance-aggressiveness and average trait Dirty Dozen.17/.14, arrogance-aggressiveness and trait narcissistic rivalry.15/.05, see also [109], for similar procedures).

### Method CONNECT

To directly study the getting-to-know process in a real-world setting, we complemented the laboratory-based approach realized in PILS by a field-based approach: CONNECT. This Method section provides an overview of the participants, procedures, and measures used in CONNECT. Further details can be obtained in the codebook of this study available under osf.io/2pmcr [86].

### Participants CONNECT

Potential participants were all psychology freshmen starting at the University of Münster, Germany, in October 2012 (*N =* 138; maximum possible sample size). They received an invitation via email to participate in the study and to attend an introductory session 1 week before the start of the semester. More than 90% of the students agreed to participate, resulting in a sample of 131 participants (107 female). However, five participants dropped out of the study, leaving a final sample of 126 participants (105 female). They were between the ages of 18 and 42 years (*M* = 21.35, *SD* = 3.96) and participated in exchange for research participation credit, monetary compensation (up to 260 Euro), participation in a lottery for a variety of gift vouchers, and individual feedback on their personality and personality development. This study’s sampling strategy was based on the actual size of the cohort of students. Because a total number of 138 freshmen started their psychology studies that fall, this number defined the maximum possible sample size. All procedures of this study were approved by the review boards of the University of Mainz and the University of Münster and are in line with the recommendations of the DFG and DGPs.

### Overview CONNECT

We collected five types of assessments to capture the development of personality and social relationships in our sample from the very first time the participants met onwards (see Table 9).

**Table 9:** Number of participants per data source.

	*N*	Women	Men
**Zero-acquaintance experiment**			
Introductory session	109	85	24
Late starters	17	16	1
**Online survey**			
T1 — Self-report	124	101	23
T1 — Informant-report	120	97	23
T2 — Self-report	106	85	21
T3 — Self-report	108	90	18
T4 — Self-report	99	84	15
T5 — Self-report	92	75	17
**Time-based assessment**			
Diary 1 to 7 (Phase 1)	124	100	24
Diary 8 to 15 (Phase 2)	121	98	23
Diary 16 to 21 (Phase 3)	109	87	22
Diary 22 (Phase 4)	97	82	15
Diary 23 (Phase 5)	89	73	16
**Event-based assessment**			
Phase 1	119	95	24
Phase 2	102	82	20
Phase 3	88	70	18
**Laboratory assessment**	92	74	18
**Total**	126	101	25

*Note*. For the time-based assessment and the event-based assessment, participants were counted when they reported at least one diary/one interaction in the corresponding phase.

**Fig 4:**
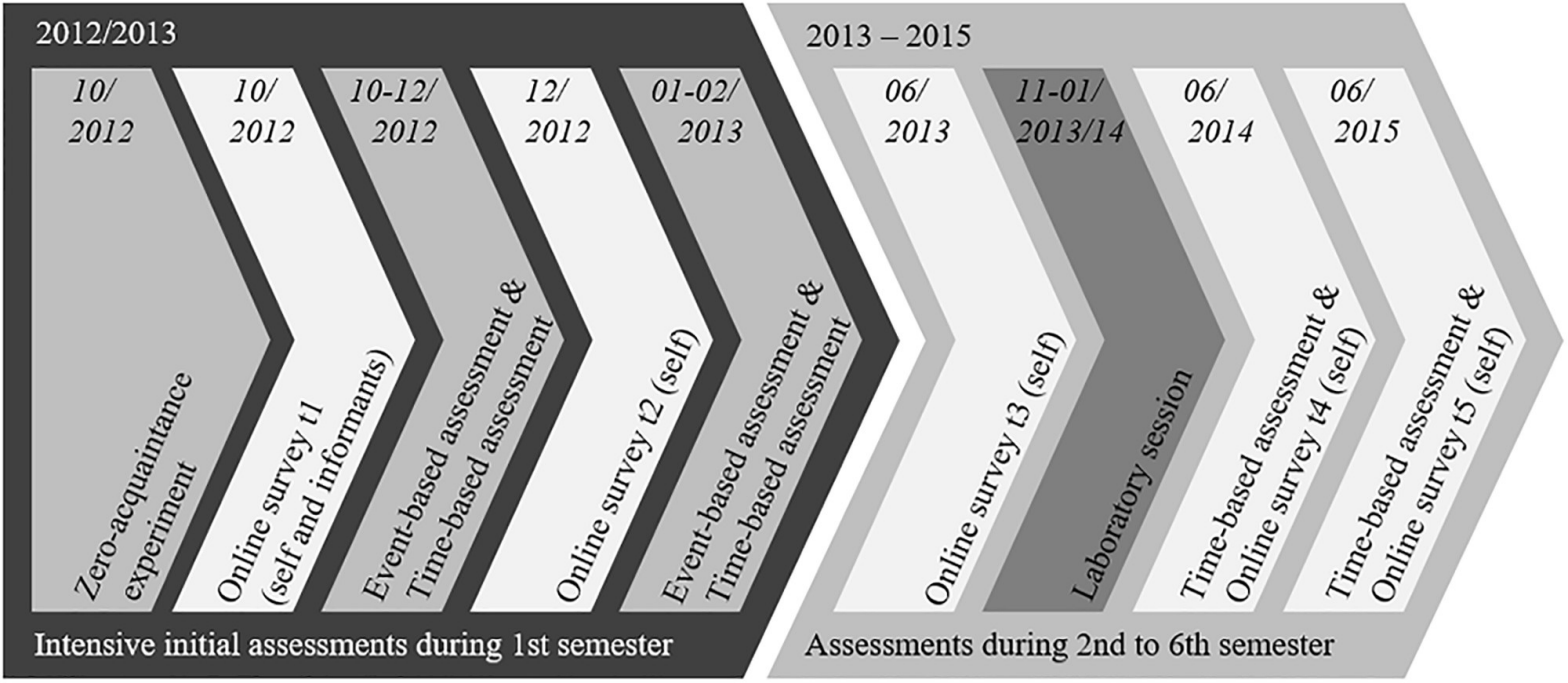
CONNECT’s Timeline.

The study began with an introductory session including a zero-acquaintance experiment to capture mutual first impressions, representing the first type of assessment. The second type of assessment involved online surveys to measure participants’ demographics and traits. This included five assessments of personality self-reports as well as an informant-report at the beginning of the study. The third type of assessment involved a time-based online diary to obtain social-relationship indicators and interpersonal perceptions at regular time intervals. The fourth type of assessment was event-based. Across 5 weeks of their first semester, participants were asked to complete a smartphone-based survey after social interactions with fellow students to capture behavioral, intra- and interpersonal perceptions, as well as state-affect ratings during real-life social interactions. The fifth type of assessment involved direct observations in laboratory-based experimental sessions (approximately one year after the beginning of the study). This standardized setting allowed us to collect physical, behavioral, and cognitive data that could not be collected in the field. A schematic overview of the different types of assessments across the duration of the study can be found in Fig 4. In the following, we will describe the procedures and measures used for each type of assessment.

### Procedure CONNECT—Zero-acquaintance experiment

The introductory session took place at the beginning of the winter semester (i.e., 1 week before classes started). A total of 113 participants attended the introductory session. At the beginning of the session, participants received a button with their randomly assigned respondent number (CONNECT number) and were asked to visibly wear the button. In addition, participants received a folder with their CONNECT number including a questionnaire booklet, an informed consent form, and a manual explaining the study. The CONNECT numbers on the buttons matched the seat numbers in the lecture hall in which the session took place. Participants were asked to take their designated seat in the room. After an introduction to psychology, their classes, and a short introduction to the study, participants were asked to complete a brief state-affect questionnaire and to subsequently introduce themselves. Beginning at the right-hand side of each row, participants were requested to individually step forward to a marked spot on the floor and briefly introduce themselves by stating their CONNECT number, first name, age, and place of origin. All self-introductions were videotaped with a camcorder (Sony HDR-XR106E with Sony Wide Conversion Lens x0.7, type VCL-HA07A). After each introduction, participants were evaluated by all other freshmen with regard to interpersonal perceptions on a paper-and-pencil rating sheet. Thus, we used a round-robin design in which every participant was rated by and rated every other participant [49]. After each introduction, a full-body picture and a portrait picture were taken of each participant (using two digital cameras). Participants were asked to stand still and to look into the camera with a neutral facial expression (i.e., neither positive nor negative). Following the evaluation, participants in the respective row all moved one seat to the right, and the evaluated participant took the empty seat at the far left-hand side of the row. This procedure was repeated row by row until all students were rated (see [70,71,130], for the implementation of a similar procedure). After the introductory session, participants again completed the same brief state-affect questionnaire as in the beginning of the session.

After the zero-acquaintance experiment, participants were introduced to the subsequent procedures and assessment types of the study. The event-based and time-based assessments were explained in more detail to ensure that all participants were able to complete the online diaries and the smartphone-based survey. Participants who did not possess a smartphone were equipped with an iPod Touch for the duration of the event-based assessment. During the information session, the portrait pictures of all participants were properly formatted and uploaded into the smartphone-based survey. The zero-acquaintance assessment finished with an informal gathering in which the participants had the opportunity to get to know each other and to start using the smartphone-based survey (i.e., to evaluate interactions with fellow students; see event-based assessment below).

A total of 17 participants who were not able to attend the introductory session attended individual late-starter meetings. During these meetings, participants received all information that was provided during the introductory session. In addition, they provided the same self-introductions, which were videotaped. Portrait and full-body pictures were taken as was done for the other participants in the introductory session. However, their self-introductions were not evaluated by their fellow students. With the exception of the zero-acquaintance round-robin evaluations, the procedure of data collection was identical for late starters and participants who attended the introductory session.

### Measures CONNECT — Zero-acquaintance experiment

#### Physical and behavioral measures

Trained raters underwent a training procedure that was identical to the one used in PILS. This involved the physical and behavioral assessments regarding the zero-acquaintance experiment as well as the laboratory session (for ICCs see Results section). Again we provide an overview of physical appearance and behavioral ratings that have already been obtained. This rich physical and behavioral data base has yet to be tapped for additional ratings and codings.

**Physical appearance ratings:** As in PILS, the portrait and full-body photographs were each rated by trained coders on 4 items, respectively (portrait: participants’ facial attractiveness, hardness (as opposed to babyfaceness), styled hair, neat/well-groomed hair; full-body: participants’ attractiveness of the body, flashy clothing, neat/well-groomed clothing, modern clothing). All attributes were rated on scales ranging from 1 (*not at all*) to 10 (*very much so*).

**Individual behavioral assessments:** The video footage was rated by trained coders on five global behavioral dimensions on a 6-point scale ranging from 1 (*not at all*) to 6 (*very much so*): friendly behavior (i.e., offers an explanation for a better understanding, behaves politely, behaves kindly), expressive behavior (i.e., shows positive emotions, speaks a lot, behaves actively, expressively), negative affect/nervousness (i.e., shows tense, inhibited behavior, behaves insecurely, anxiously), dominant/self-confident behavior (i.e., behaves self-confidently, has a strong presence, behaves self-assuredly, behaves powerfully), and arrogant behavior (i.e., shows cocky, bigheaded behavior, behaves in a braggy, arrogant way).

#### Interpersonal perceptions

Interpersonal attraction: Participants provided ratings of liking (“How much do you like this person?”) and metaperceptions of liking (“How much does this person like you?”) for all fellow students on scales ranging from 0 (*not at all*) to 5 (*very much so*).

**Personality impressions:** Participants judged and were judged by all other participants with regard to dominance (rated from 0 = *submissive/insecure* to 5 = *dominant/self-confident*) and affectionateness (rated from 0 = *cold-hearted/manipulative* to 5 = *loving/trustworthy*).

#### Relationship indicators

Emerging social relations: Participants judged and were judged by all other participants with regard to acquaintance (0 = *unacquainted*, 1 = *seen once*, 2 = *already exchanged a few words*, 3 = *already talked for a while*, 4 = *known for some time* to 5 = *good friends*).

#### Self-perceptions and state affect

State affect and state self-esteem: Participants rated their state affect and state self-esteem on bipolar scales ranging from 1 to 7. The following five states were assessed with the anchors: good versus bad mood, bored versus activated, nervous versus relaxed, inhibited versus determined, and satisfied versus unsatisfied with myself.

### Procedure CONNECT — Online survey

One day after the introductory session, participants received an invitation to an online survey via email. At the end of that questionnaire, participants were asked to again provide their email addresses to receive an automatized email with a personalized link to an online informant-rating survey. Participants were asked to send the link of the informant survey to (at least) two family members or well-acquainted friends. The informant version of the questionnaire consisted of measures that were identical to the self-report version with the exceptions of information regarding social networks, which were not assessed in the informant-report, and the use of shortened versions of the Narcissistic Personality Inventory and Sexual Orientation Questionnaire. Online self-report surveys were completed at five time points, that is, at the beginning of the study (October 2012; T1), during Christmas break (December 2012; T2), at the end of the second semester (June 2013; T3), during the fourth semester (May 2014; T4), and during the sixth semester (June, 2015; T5). Self-reports were obtained at all waves, whereas informant-ratings were assessed only at T1. The number of informant-reports per participant ranged from one to three (*M*_no_ = 2.04; *SD*_no_ = 0.52; *N*_total_ = 247; 175 female; *M*_age_ = 28.59; *SD*_age_ = 14.41). All surveys (T1 to T5) included questions with regard to demographic information, online social network use, the participants’ broader social network, and an extensive battery of personality trait questionnaires. At T3, T4, and T5, the online survey was supplemented by assessments of a number of retrospective questions, exams taken, and grades obtained during participants’ studies. Moreover, additional demographic information and a selection of various life events were assessed at T5.

### Measures CONNECT — Online survey

Most of the measures in the CONNECT online survey were similar or identical to the measures assessed in PILS. Therefore, we describe in detail only the measures that were different from PILS. Otherwise, we simply indicate parallelism. For descriptive results and reliabilities, please refer to Results section.

#### Demographic information

Demographic information was identical to the assessment in PILS. Additional questions involved the name of the orientation week group at the beginning of the first semester (only in T1), the living situation, and information about participants’ course of studies.

#### Trait measures

Similar to PILS, we assessed the Big Five, self-concept, self-esteem, need to belong, trait affect, narcissism, the dark triad, impulsivity, anger, sensation seeking, shyness and sociability, and sociosexual as well as sexual orientation. The trait measures in the online survey differed from the one used in PILS with regard to an extension of the trait affect scales as well as the self-concept measure and the inclusion of communal narcissism.

**Trait affect:** The four items used in PILS were supplemented by three items referring to “in a good mood,” “nervous/jittery,” and “relaxed.”

**Self-concept:** The 23 items used to measure participants’ self-concepts in PILS were extended to a total of 29 self-concept items in the CONNECT trait assessment. We used six additional items for a more detailed assessment of interpersonal aspects of participants’ self-concepts (i.e., “dominant,” “insecure,” “affectionate,” “cold-hearted,” “arrogant,” “exploiting”). The response format was identical to the one used in PILS; however, here, the CONNECT cohort served as the reference group.

**Communal narcissism:** Communal narcissism was assessed using a selection of seven items of the 16-item Communal Narcissism Inventory (CNI; [131]). Selected items were “I will be well known for the good deeds I will do,” “I am the most caring person in my social surroundings,” “I greatly enrich others’ lives,” “I will bring freedom to the people,” “I have a very positive influence on others,” “I am generally the most understanding person,” and “I’ll make the world a much more beautiful place.” Items were answered on 7-point scales ranging from 1 (*absolutely false*) to 7 (*absolutely right*).

#### Interpersonal perceptions

Interpersonal attraction: Retrospective reports on metaperceptions of liking were obtained at T3, T4, and T5 by asking participants to rate the extent to which their fellow students liked them at the current and all previous time points of the online survey. These items were rated on 11-point scales ranging from 1 (*dislikeable/they were annoyed by me*) to 11 (*likeable/liked me*).

**Retrospective accuracy perceptions:** At T3, T4, and T5, participants were asked to retrospectively rate (a) their own accuracy of interpersonal perceptions (“How well could you judge your fellow students at the beginning of the first semester?”), (b) their fellow students’ accuracy (“How well could your fellow students judge you at the beginning of the first semester?”), and (c) their knowledge of their fellow students’ perceptions of them (“At the beginning of the first semester, how well did you know how your fellow students judged you?”). Furthermore, from T3 onwards, participants reported on these items for the current time point and all previous online survey time points. Participants answered the items using an 11-point slider ranging from 1 (*0%*) to 11 (*100%*).

#### Relationship indicators

Social interactions: Participants retrospectively provided four general ratings of their overall interactions with fellow students at T3, T4, and T5. They were asked whether the interactions were interesting versus uninteresting (positive versus negative) and whether they thought their interaction partners found the interactions to be interesting versus uninteresting (positive versus negative; e.g., “On average I think the interactions with my fellow students were…”). Items were answered on bipolar rating scales ranging from 1 (*interesting*) to 7 (*uninteresting*) or 1 (*positive*) to 7 (*negative*), respectively.

**Emerging social relationships:** Participants were asked to retrospectively report on their experiences in getting to know fellow students and developing friendships with fellow students at T3, T4, and T5. Therefore, participants completed nine additional items (“I found it difficult to get to know my fellow students,” “I did well in making new friends with my fellow students,” “It was easy for me to approach my fellow students and be an inspiration to them,” “My opinion is very important to my fellow students,” “More of my fellow students should be friends with me,” “Sometimes I’m really proud that so many of my fellow students want to be friends with me,” “Sometimes I’m disappointed that not more of my fellow students want to be friends with me,” “Sometimes I’m ashamed that not more of my fellow students want to be friends with me,” “Sometimes I almost feel guilty that not more of my fellow students want to be friends with me”) on 6-point scales ranging from 1 (*do not agree at all*) to 6 (*agree completely*). Furthermore, from T3 onwards, participants reported the number of fellow students they would call friends and the number of fellow students who would consider them to be a friend at the current time point and at all previous online survey waves (except for T1).

**Relationship quality:** Participants were asked to rate the general quality of their relationships with close friends who were also fellow students and additional friends and acquaintances who were also fellow students at T2, T3, T4, and T5. They gave the number of members of each respective group and rated their frequency of contact on 5-point scales ranging from 1 (*daily*) to 5 (*once a month or less*) as well as questions regarding the quality of those relationships. Specifically, participants rated relationship characteristics, such as relationship satisfaction (e.g., “With respect to my relationships with my close friends who are also my fellow students, I am …”) on a scale ranging from 1 (*very dissatisfied*) to 7 (*very satisfied*), relationship importance from 1 (*not important*) to 7 (*very important*), conflicts from 1 (*never conflicts*) to 7 (*conflicts each time we meet*), emotional support from 1 (*I never turn to my close friends who are also my fellow students*) to 7 (*I always turn to my close friends who are also my fellow students*), exchange ideas of interest from 1 (*not discuss at all*) to 7 (*very good discussion*), and acceptance (instrumental support) from 1 (*not accepted at all*) to 7 (*completely accepted*).

**Broader social network:** Information about participants’ broader social network was obtained by assessing participants’ social relationships outside the college context. First, they specified the number of people in each relevant group (e.g., core family, broader family) and then rated the frequency of contact on 5-point scales ranging from 1 (*daily*) to 5 (*once a month or less*). Second, the same items that were used to assess relationship quality were presented with regard to participants’ relationships with their core family (mother, father, siblings, excluding self), broader family (all family members outside the core family), romantic partner, other romantic relationships, close friends who were not fellow students, and additional friends and acquaintances who were not fellow students at all five time points (T1 to T5).

#### Additional life event and outcome measures

Online social network characteristics: At all time points, we used eight items to obtain data on participants’ social network use. These were questions about the numbers of profiles in social networks, hours spent on favorite social networks per week, friends, groups, wall posts per week, photo albums, photos in which the subject is tagged, and pages that the subject has “liked.”

**Academic achievements:** At T5, participants were asked to provide their average high school graduation grade as well as their high school graduation subjects. Another question asking whether they had worked as research assistants at the university during the last six semesters was also added at T5. At T3, T4, and T5, they provided further information on the exams taken and the grades obtained during the last six semesters on the German grading scale ranging from 1.0 (*excellent*) to 1.3, 1.7, 2.0, 2.3, 2.7, 3.0 3.3, 3.7, and 4.0 (*sufficient*) and 5.0 (*insufficient*). At T5, participants were further asked to provide information regarding whether or not they (a) had passed all relevant exams and, if so, (b) if they had passed an exam at their first, second, or third attempt. To receive additional information from the examination office about other grades, participants were asked to provide their student identification number.

**Life events:** At T5, a selection of 63 specific life events, adopted from TOSCA [132] and the Life Experience Survey (LES; [133]), as well as some self-selected life events, were added to obtain additional information about participants’ lives prior to and during the course of their studies (see S3 Table). Life events concerned the categories “family” (5 items, e.g., “family member was seriously ill or injured,” “parents got divorced”), “friends” (5 items; e.g., “close friend died,” “made a new friend”), “romantic relationships” (17 items, e.g., “began a new romantic relationship,” ”partner was seriously ill or injured”), “personal life” (23 items, e.g., “suffered from serious illness/injury,” “had personal success”), “work” (5 items, e.g., “started new job,” “significantly increased working hours”), “studies” (8 items, e.g., “changed my major,” “did not pass exam”), and one open category to indicate other events that participants may have experienced and valued as meaningful. For each life event, participants were first asked whether or not it had happened during the last 3.5 years (i.e., in the last 6 months prior to the start of their studies and during their studies). If yes, they were then asked to name the exact half-year period (seven options), starting 6 months before the official start of their psychology studies. When participants had experienced a life event more than once, a multiple selection of time periods was possible, and the questions that followed were asked for each time period separately. Third, they were then asked to specify the exact month when the life event had happened. Fourth, they were asked to judge the subjective impact of the life event on a 5-point scale ranging from 1 (*very negative*) to 5 (*very positive*). Fifth and last, with an open-response format, they were asked to briefly describe what had happened. If participants indicated that the life event did not happen to them (under “first”), all further questions (from “second” to “fifth”) were omitted.

**Self-perceived personality development:** At T3, T4, and T5, participants were asked to rate their own personality development and the personality development of their fellow students. They were asked to what degree (a) they themselves and (b) their fellow students had changed on the Big Five traits, on dominance, interpersonal warmth, narcissistic admiration, and narcissistic rivalry since T1 (at T3), since T3 (at T4), and since T4 (at T5). They answered these items using a slider control that ranged from 1 (*greatly decreased*), 2, 3, 4, 5, 6 (*unchanged*), 7, 8, 9, 10, to 11 (*greatly increased*).

**Further individual information:** At T5 only, we assessed specific variables such as height, weight, and handedness, the average time participants got up and went to bed on workdays, average yearly sick days, favorite subjects at school, maximum length and quantity of romantic relationships, frequency of going out, life satisfaction, experiences abroad, language skills, and participants’ smoking and alcohol drinking behavior.

### Procedure CONNECT — Time-based assessment

Three versions of individualized online diaries were used to obtain a time-based assessment of relationship indicators, interpersonal perceptions, and self-perceptions. Version A of the diary involved the assessment of acquaintance, liking, and metaperceptions of liking of fellow students. Version B contained questions with regard to social status and personality impressions of interaction partners in the previous week. In Version C, participants provided information with regard to the quality of their relationships with their interaction partners in the previous week. Both Versions B and C were always preceded by Version A. That is, participants filled out either a short version of the diary (only Version A) or one of two long versions of the diary (Version A+B, or Version A+C).

In the first 2 weeks, participants completed two short diaries (Version A) during the week and one long diary (Version A+B or A+C) over the weekend, respectively. Versions A+B and A+C were alternated so that participants completed each long diary version every other week. From the third week of the study until the end of the semester (4 months after the beginning of the study), participants completed either Diary A+B or Diary A+C once a week. Additional follow-up assessments were administered along with the online surveys at T4 and T5, involving diary Versions A+B+C. In summary, a total of 23 diaries were assessed, with seven diaries consisting of only Version A, seven of Version A+B, seven of Version A+C, and two (at T4 and T5) of a complete version involving Diary A+B+C.

### Measures CONNECT — Time-based assessment

#### Interpersonal perceptions: Interpersonal attraction

Participants provided ratings of liking (“I find this person…”) and metaperceptions of liking (“This person finds me…”) for all fellow students on scales ranging from -5 (*unlikeable/annoying*) to 5 (*likeable/nice*) in each online diary (online diary Version A).

**Status perceptions:** Participants were asked to sort photographs of all of their fellow students with regard to their social status within the cohort (online diary Version C). This resulted in a ranking sequence from 1 (*highest status*) to 130 (*lowest status*). From T4 on, the status assessment as well as the self-perceived status was moved from the diary into the online survey using a simplified rating procedure. Here, the photographs and names of all participants, including the person answering the survey, were shown in a random order, and participants dragged and dropped these photographs into five status groups: (a) very low (5 persons), (b) low (10 persons), (c) intermediate (all other persons not included in a, b, d, and e), (d) high (10 persons), and (e) very high (5 persons). The default grouping (i.e., not moving a photograph by dragging and dropping) of all persons was Group c so that participants were only to move the 30 persons possessing very low, low, high, and very high status into the respective groups (respective files at the bottom of the page by dragging and dropping). The resulting ranks of fellow students were saved as indicators of perceived status. Status was described as the importance, recognition, and influence a person had within the social group of freshmen.

**Personality impressions:** Based on a question targeting with whom of their cohort participants had interacted within the past week, they rated their respective last-week interaction partners with regard to 11 characteristics on 11-point bipolar scales (Diary B): dominance (from dominant, self-confident to submissive, insecure), affectionateness (from affectionate, trustworthy to cold-hearted, manipulative), extraversion (from extraverted, enthusiastic to reserved, quiet), agreeableness (from critical, combative to understanding, warmhearted), conscientiousness (from reliable, self-disciplined to unorganized, careless), emotional stability (from anxious, easy to upset to relaxed, emotionally stable), openness to experience (from widely interested, profound to conventional, uncreative), intelligence (from intelligent to unintelligent), physical attractiveness (from physically attractive to physically unattractive), narcissistic admiration (“This person is narcissistic and wants to be admired”), and narcissistic rivalry (“This person does not think much of others, wants to be better than others”), both answered from 0 (*do not agree at all*) to 10 (*agree completely*).

#### Relationship indicators

Emerging social relations: We measured the emergence of new social relationships with three kinds of measures. First, participants judged and were judged by all other participants with regard to acquaintance (1 = *unacquainted*, 2 = *seen once*, 3 = *already exchanged a few words*, 4 = *already talked for a while*, 5 = *known for some time* to 6 = *good friends*; diary Version A). Second, participants’ frequency of interactions with fellow students was assessed by asking participants to identify fellow student with whom they had interacted in the previous week by selecting their photographs from a scroll-down menu and to report the number of interactions with each interaction partner (online diary Versions B and C). Third, participants selected fellow students with regard to three relationship roles (online diary Version C): friends (“This person is a good friend of mine…”), leaders (“This person is a good leader…”), potential dates (“I can imagine going on a date with this person…”). These items were answered with a forced choice of “yes” or “no.”

**Relationship quality:** Participants provided ratings with regard to the quality of the social relationships for all fellow students with whom they had interacted during the previous week (diary Version C). Specifically, participants rated relationship satisfaction (“I am… with my relationship with this person”) on a scale ranging from 0 (*very unsatisfied*) to 10 (*very satisfied*), relationship importance (“My relationship with this person is … to me”) ranging from 0 (*not important*) to 10 (*very important*), conflicts (“I have had… with this person”) from 0 (*no conflicts*) to 10 (*conflicts at every occasion*), emotional support (“When I have emotional troubles, I can…”) ranging from 0 (*never turn to this person*) to 10 (*always turn to this person*), instrumental support (“When I have trouble at university, I can…”) ranging from 0 (*never turn to this person*) to 10 (*always turn to this person*), exchange ideas of interest (“I can exchange ideas with this person about topics that are important to me [e.g., sports, hobbies, music, politics, culture, science]”) ranging from 0 (*cannot exchange at all*) to 10 (*can exchange very well*), and acceptance (“I feel … by this person”) ranging from 0 (*not accepted at all*) to 10 (*entirely accepted*).

**Broader social network:** In addition (diary Version A), participants were asked to provide information about the number of people they had interacted with who were not their fellow students. Also, they were asked to estimate the percentage of time spent interacting with others who were not fellow students during the previous week on a slider ranging from 0% to 100%.

#### Self-perceptions and state affect

Self-liking: Participants rated how much they liked themselves in diary Version A on a scale from 0 (*unlikeable*, *annoying*) to 10 (*likeable*, *nice*).

**Personality self-impressions:** In online diary Version B, participants rated themselves on the same 11 characteristics they rated their fellow students on (dominance, affectionateness, extraversion, agreeableness, conscientiousness, emotional stability, openness to experience, intelligence, physical attractiveness, admiration, and rivalry).

**Self-perceived status:** During the sorting of their fellow students according to social status (online diary Version C, described above), participants also sorted their own picture, resulting in an additional measure of self-perceived social status. Thus, participants rated their own current social status within the group of freshmen at seven time points during the first semester, whenever information of diary Version C was obtained. At T4 and T5, also the self-perceived status was moved from the online diary to the online survey (see status perception for details of procedure).

### Procedure CONNECT — Event-based assessment

We measured event-based ratings of self-perceptions, interpersonal perceptions, as well as relationship indicators in real-life social interactions by means of an online questionnaire for smartphones. There were three phases of event-based assessment. The first phase took place in the first 3 weeks of the study, starting at the informal gathering after the zero-acquaintance experiment. The second phase took place 2 months later (1 week, beginning of December 2012) and the third phase another 6 weeks later (1 week, end of January 2013).

Within each of these 5 weeks of event-based assessments, participants were asked to report on every interaction they had with a fellow student. An interaction was defined as an encounter with one or more people that lasted at least 5 minutes and in which one responded to the behavior of the other persons (see [134–136] for a similar procedure). Immediately after each interaction, they had to report the number of interaction partners, the duration of the interaction, and the situation category (typical situation for freshmen, e.g., meeting at class, at lunch, or in a bar). Subsequently, participants were asked to select their interaction partners using a scroll-down menu of pictures (portrait picture made at the introductory session, along with the CONNECT number and first name). They were then requested to judge their state affect, their own behaviors, and the behaviors of each interaction partner during the interaction. Finally, they also provided an evaluation of the interaction.

A paper-and-pencil version of the smartphone-based survey was provided for cases in which participants did not have internet access on their smartphone or iPod Touch after an interaction. The offline version consisted of a post-it block containing the same questions as the smartphone-based survey. A CONNECT mailbox was available in the main Psychology building for participants to anonymously hand in their offline ratings.

### Measures CONNECT — Event-based assessment

#### Physical and behavioral measures

Interactional behavioral ratings: Participants rated their own behavior and the behavior of each of their interaction partners during the interaction with regard to seven behaviors (“Please judge your own behavior,” “Please judge his/her behavior”): Dominant versus submissive, sociable versus reclusive, friendly versus unfriendly, arrogant versus modest, exploiting versus cooperative, self-revealing versus reserved, reliable versus unreliable. All items were answered on bipolar scales ranging from 1 to 7.

**Situation selection:** The participants reported the duration (in minutes) of the interaction and the situation in which the interaction took place. They could choose from 17 options: at university during class, at university outside class, at university in bistro/canteen, studying/university-related activities, meeting/conversation at home, meeting/conversation outside, meeting/conversation in restaurant, short message/email/Facebook, phone call, chance meeting, celebration/party, television/DVD/cinema, cultural activities, shopping, sports, romantic activity, and volunteering.

#### Relationship indicators

Social interactions: Participants provided four general ratings of interactions in the event-based assessment. They were asked whether the interaction was interesting and positive and whether they thought their interaction partner would evaluate the interaction as interesting and positive (meta-evaluation). Items were answered on bipolar rating scales ranging from 1 (*interesting*) to 7 (*uninteresting*) or 1 (*positive*) to 7 (*negative*), respectively.

**Emerging social relations:** The event-based assessments also provided us with general information about the number of real-life interactions with fellow students as well as the number of interaction partners within each interaction.

#### Self-perceptions and state affect

State affect and state self-esteem: Participants rated their state affect and state self-esteem after every reported interaction on bipolar scales ranging from 1 to 7. The following six items were assessed: good versus bad mood, bored versus activated, satisfied versus unsatisfied with myself, nervous versus relaxed, inhibited versus determined, and satisfied versus unsatisfied with the interaction.

### Procedure CONNECT — Direct observations

Approximately one year after the start of the study (November 2013 to January 2014), participants were invited to take part in a laboratory session. Four participants were invited for every time slot. After signing an informed consent form, participants (*N* = 92) received general instructions regarding the procedure. Specifically, they were told that the laboratory session would consist of four parts (see Table 10): One part in the video laboratory and three parts in the computer laboratory. On the basis of their registration in the laboratory sessions, participants were assigned to a specific order in which they completed the four parts of the laboratory-based assessments. Participants rotated through the video laboratory so that at each time slot (about 20 min), three participants worked on tasks in the computer laboratory, while one participant was observed in the video laboratory. During the short breaks between each of the four parts, two participants stayed in the computer laboratory while the other two changed from one laboratory to the other.

**Table 10:** Overview of Direct Behavioral Assessment Situations in CONNECT.

Situation	Task description	Average duration
**Zero-acquaintance experiment**		
Self-Introduction 1	Each participant stood in front of the other participants and mentioned her/his CONNECT number, name, age, and hometown. During the self-introduction, all remaining participants made five judgments about the participant up front.	10 sec
**Laboratory-based assessment**		
Small talk	The experimenter asked the participants the following standardized questions: “How are your studies going?”, “What subjects do you like the most?”, and “Are you getting along with your fellow students?”.	1 min
Self-Introduction 2	Participants stood on a marked position and gave a brief self-introduction while speaking to cameras at the opposite end of the room. The proposed topics were the participants’ hobbies and interests.	3 min
Stress test	Participants prepared and gave a videotaped speech about an unknown text on the topic “blood.”	3 min
Public Goods Game	Every group member received 5€ in 50-cent coins and decided how much money she/he would take for her/himself and how much she/he would deposit into a common group pot/common pot for all participants.	10 min

#### Video laboratory

**Fig 5:**
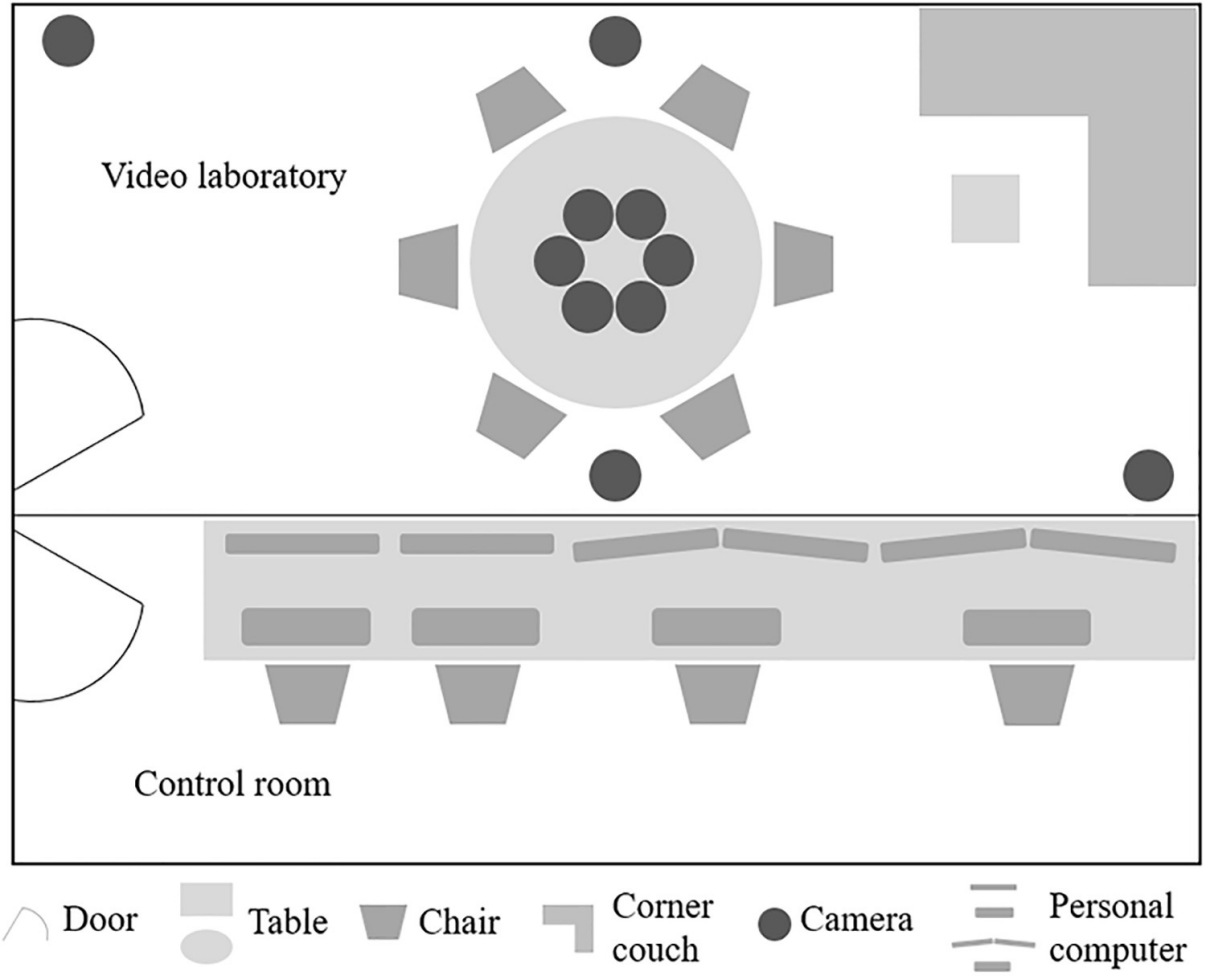
Schematic overview of the video laboratory and control room in CONNECT.

The setup of the video laboratory was almost identical to the video laboratory in PILS (see Fig 5).

In addition to the setup used in PILS, the video laboratory in CONNECT was also equipped with a corner couch and a glass table located opposite the door in the left corner of the room. Video recording had been started before the participant entered the room, and his/her behavior was recorded throughout all tasks (see Table 10 for an overview). Before leaving for the control room, the experimenter had wired the participant with a microphone on a corner couch and simultaneously engaged the participant in standardized small talk (asking: “How are your studies going?”; “What subjects do you like the most?”; “Are you getting along with your fellow students?”; see [130], for a similar procedure). Afterwards, the participant was asked to give a self-introduction in front of the camera next to the corner couch and to tell something about him- or herself within 3 min (“Please tell us something about yourself, about your hobbies, what you are interested in, and so on. You have 3 minutes. Alright? Then you can start now.”). When participants could not fill the 3 min, they were further instructed by the experimenter (“You still have time”; “Can you think of something else?”; “Think about it again”), and they were stopped when they reached the 3-min mark (“I need to ask you to finish now”). After finishing the self-introduction, participants were confronted with a stress test (see [137], for a successful previous application of this task). In this stress test, participants were instructed that the goal of the test was to examine the degree to which they were able to comprehend and reproduce a scientific text under time pressure. They were told that this ability would be very important for successfully completing their psychology degree and that they would have 4 min to read an intellectually challenging text that had been adapted from a physiology textbook about the composition and function of blood [138]. Subsequently, participants were requested to reproduce the knowledge they had learned in a 3-min presentation, which was videotaped.

In the final part in the video laboratory, participants completed three economic tasks for the assessment of prosocial behavior: two versions of the Public Goods Game [139–141], followed by the social value orientation (SVO; [142–144]) questionnaire. In all tasks, participants were asked to make choices among combinations of outcomes for their own good or for the collective good. However, the three tasks varied with respect to how personally salient versus abstract the social reference group was presented―the group toward which participants could behave more or less prosocially. This ranged from a highly specific reference group (all four participants from the same laboratory session), to a less specific but still concrete reference group (all participants in CONNECT), to a highly abstract social reference (fictitious unknown other person in the SVO questionnaire). The participants played these games on the glass table when seated on the corner couch in the video laboratory. In the first game, participants received 5 Euro in 50-cent coins and could decide how much they wanted to keep for themselves and how much they wanted to put into a public pot. They were told that the other three participants in the session would make the same decisions and thus would also put money in the public pot. They were further told that all money in the pot would then be doubled by the experimenter and equally distributed among the group members. Participants were further informed that (a) a higher contribution to the collective pot would result in a higher benefit for the entire group, (b) they would also receive a quarter of the group money even if they did not at all contribute to the public pot, (3) they could keep the money they did not contribute to the public pot, and (4) the other participants would not know how much each person had deposited in the public pot. After having received these instructions, participants were to put the money in individual envelopes. In the second game, the same game was played again, but this time, the collective good represented not only the four participants in the session but the whole CONNECT cohort (i.e., the total number of participants). Finally, participants filled out the SVO questionnaire. At the end of the video laboratory session, two photographs of each participant were taken. They were then paid, thanked, and debriefed.

#### Computer laboratory

The three parts of the computer laboratory session involved, in the following order, (a) a working memory capacity task and a vocabulary knowledge task, (b) a reasoning test, and (c) Implicit Association Tests (IATs) for the indirect assessment of personality measures. All tasks were administered on a personal computer with version 4.0.2 of the program Inquisit [116]. Participants sat in separate cubicles.

### Measures CONNECT — Direct observations

#### Trait measures

As in PILS, we measured reasoning, vocabulary knowledge, and working memory capacity. Furthermore, we assessed implicit personality and SVO.

**Implicit personality:** We measured implicit personality during the laboratory-based session using IATs [145] to assess self-esteem, extraversion, neuroticism, and agreeableness [130,146,147]. For each of the four IATs, we followed the standard procedure for the administration and scoring of IATs. Participants were asked to sort stimuli representing four concepts (“me,” “others,” Trait Pole 1, e.g., “extraversion,” Trait Pole 2, e.g., “introversion”) using just two responses (represented by two keyboard keys), each assigned to two of the four concepts. A participant’s score for each of the traits (e.g., extraversion) was estimated by comparing the mean reaction time in the blocks in which the self-category (“me”) was paired with one trait-pole category (e.g., “extraversion”) with the mean reaction time in blocks in which the self-category was paired with the other trait-pole category (e.g., “introversion”). IAT data were treated with an improved scoring algorithm (the so-called D1 measure) as described by Greenwald, Nosek, and Banaji [148]. To minimize fatigue and the utilization of response strategies, participants took breaks between each of the four IATs, during which they were presented with one thematic apperception test (TAT) picture and instructed to write down their ideas concerning the situation displayed in the picture (see [130,147].

#### Physical and behavioral measures

Physical appearance ratings: The portrait and full-body photographs taken during the laboratory sessions were rated by trained coders on eight items: participants’ facial attractiveness, hardness (as opposed to babyfaceness), styled hair, neat/well-groomed hair (all based on the portrait picture), attractiveness of the body, flashy clothes, neat/well-groomed clothes, modern clothing (all based on the full-body picture). All attributes were rated on scales ranging from 1 (*not at all*) to 10 (*very much so*).

**Individual behavioral assessments:** The video footage was rated by trained coders for each of the different parts of the sessions, namely, (a) small talk (i.e., warmth, expressiveness, nervousness, arrogance), (b) self-introduction (i.e., expressiveness, nervousness, self-confidence, arrogance, warmth), and (c) stress test (i.e., nervousness, intellectual ability, expressiveness, self-confidence). All behaviors were rated on scales ranging from 1 (*not at all*) to 6 (*very strong*).

Video footage of small talk in the laboratory setting was rated with regard to nine behaviors in four categories: warm-heartedness (i.e., behaves thoughtfully, politely, pays attention to experimenter; smiles affirmatively, friendly, smiles back; provides positive feedback, paraphrases in a positive way, and makes affirmative statements), expressiveness (i.e., makes funny, ironic, relaxing, and humorous statements; provides a lot of personal information, is expressive, is talkative, behaves openly; shows interest in social interactions, behaves gregariously, responds sociably to others), negative affect/nervousness (i.e., feels uncomfortable; behaves negatively, pessimistically, devalues oneself with what one says), and arrogance (i.e., shows self-admiring, vain behavior, thinks of oneself as great, is self-loving).

Fourteen behaviors within five behavioral domains were rated in the self-introduction in the laboratory session: warm-heartedness (i.e., has a warm, affectionate voice; shows a friendly, affirmative facial expression), expressiveness (i.e., moves arms and hands dynamically [not nervously]; shows expressive, lively, and cheerful facial expressions; behaves actively, cheerfully, and enthusiastically; has an expressive, lively, and cheerful voice), negative affect/nervousness (i.e., stumbles insecurely or nervously in speech; has a tense, unrelaxed voice; feels uncomfortable; shows unrelaxed and tense hand gestures, shows a nervous or insecure facial expressions), dominance/self-confidence (i.e., has a powerful and loud voice; behaves self-confidently, shows a confident/dominant facial expression), and arrogance (i.e., shows self-admiring, vain behavior, adores herself/himself, is self-loving).

Four behavioral domains were rated from the video footage of the stress test: expressiveness (i.e., moves arms and hands dynamically [not nervously]; shows an expressive, lively, or cheerful facial expression; behaves actively, cheerfully, and enthusiastically; has an expressive, lively, or cheerful voice), negative affect/nervousness (i.e., stumbles insecurely/nervously, has a tense, unrelaxed voice; feels uncomfortable; shows unrelaxed, tense hand postures and gestures; shows a nervous, insecure facial expressions), dominance/self-confidence (i.e., has a powerful and loud voice; behaves self-confidently), intellectual competence (i.e., is accurate in one’s own argumentation, follows a clear logic, common thread of thoughts is always evident; is eloquent and uses articulate expressions; uses rhetorical pauses; behaves in a task- and goal-oriented way). All ratings were made on a scale ranging from 1 (*not at all*) to 6 (*very much so*; for ICCs see Results section).

**Public Goods Game and SVO:** We assessed individual differences in prosocial behavior with the two versions of the Public Goods Game. For both, the first Public Goods Game in which the collective pot was shared across the assessment group and the second Public Goods Game in which the collective pot was shared across all CONNECT participants, we recorded for each participant how many of the ten 50-cent coins they gave to the collective pot (i.e., the “public good”).

Furthermore, participants filled out the SVO questionnaire [142,143] in which they were informed that they were paired with a hypothetical random other person they did not know and would never meet in the future. They were given nine statements in which they had to choose among three combinations of points for themselves and the other person. For each choice, one of the options represented a prosocial option (e.g., “You receive 480; the other person receives 480”), whereas the other options were less prosocial and included one individualistic option (e.g., “You receive 540; the other person receives 280”) and one competitive option (e.g., “You receive 480; the other person receives 80”). We followed the standard scoring procedure and assessed for

each participant how many of the nine choices were prosocial, individualistic, and competitive.

### Results CONNECT

In this part, we provide an overview on descriptive statistics, reliabilities, and intercorrelations for all data sources assessed in CONNECT with exemplary variables for each data source. All descriptive and additional exemplary analyses with data and R Code, parallel to those presented for PILS, can be found in the online supplement of this manuscript (osf.io/zj38h/).

### Results CONNECT — Zero acquaintance experiment

In Table 11, we provide descriptive results on state-affect data before and after the zero-acquaintance experiment. After the 2-hr experiment, participants on average reported an increase in bad mood, *t*(108) = 6.23, *p* <.001, *d* = 0.60, a decrease in activation, *t*(108) = -9.97, *p* <.001, *d* = -0.96, a decrease in satisfaction with oneself, *t*(108) = -2.42, *p* =.017, *d* = -0.23, an increase in relaxation, *t*(108) = 8.26, *p* <.001, *d* = 0.79, and an increase in determination, *t*(108) = 2.85, *p* =.005, *d* = 0.27.

**Table 11:** CONNECT zero-acquaintance experiment: State affect and self perceptions.

State affect	*M*_*pre*_	*SD*_*pre*_	*M*_*post*_	*SD*_*post*_
Good mood-Bad mood	2.43	1.02	3.19	1.29
Bored-Activated	5.34	1.16	3.65	1.57
Dissatisfied-Satisfied	5.29	1.27	5.01	1.33
Nervous-Relaxed	4.03	1.51	5.28	1.42
Inhibited-Determined	4.58	1.23	4.89	1.22

*Note*. *N =* 109 perceiver. Pre = State affect before zero-acquaintance experiment. Post = State affect after zero-acquaintance experiment. Scale ranged from 1 to 7. *M* = mean between perceivers, *SD* = standard deviation between perceivers. Although late starters also provided state-affect measures, the state-affect results in this table refer to participants in the zero-acquaintance experiment only.

Table 12 displays the results of the round-robin ratings in the zero-acquaintance experiment. As in PILS and previous research [49], variance in liking judgments was for the most part a dyadic phenomenon (65% relationship variance). In contrast to PILS, however, metaliking was also found to be primarily a dyadic phenomenon (71% relationship variance) and only somewhat influenced by perceivers’ expectations (22% perceiver variance). The highest target variance (i.e., consensus) was found for perceptions of dominance/self-confidence vs. submissiveness/insecurity (i.e., dominance; 22% target variance), which mirrors the result for the assertiveness perceptions in PILS (27% target variance).

**Table 12:** CONNECT zero-acquaintance experiment. Round-robin ratings, other perceptions.

Relationship indicators & interpersonal perceptions	*M*	*Per*. *Var*.	*Tar*. *Var*.	*Rel*. *Var*.
Knowing	0.16	0.04	0.04	0.26
Liking	3.41	0.13	0.13	0.49
Metaliking	3.12	0.12	0.04	0.39
Dominance	2.98	0.10	0.18	0.54
Affectionateness	3.40	0.10	0.07	0.40

*Note*. *N =* 109 perceivers, 113 targets (due to dropout). Scales ranged from 1 to 7, except for knowing: 0 = *not at all* to 5 = *good friends*. *M* = mean aggregated across individual perceiver means, *SD* = standard deviation between perceivers. *Per*. *Var*. = Perceiver Variance, *Tar*. *Var*. = Target Variance, *Rel*. *Var*. *=* Relationship Variance.

### Results CONNECT — Online survey

Table 13 provides an overview of the trait measures assessed in CONNECT. Comparable to PILS, nearly all the Cronbach’s alpha scores were acceptable. There were, however, two low reliability scores (<.60) found for agreeableness and psychopathy. Again, the alpha values were similar to those reported in the original papers and have to be evaluated in the light of the brief nature of the measures (e.g., T1 our data/T5 our data vs. original studies: neuroticism.77/.84 vs..66, extraversion.85/.86 vs..76, openness.72/.80 vs..58, agreeableness.56/.74 vs..44, conscientiousness.71/.72 vs..60 [88]; Rosenberg Self-Esteem Scale.89/.92 vs..84 to.85 [92]; Self-Attributes Questionnaire.66/.64 vs..76 [90]; Narcissistic Personality Inventory.78/.69 vs..80 to.86 [96]; NARQ Admiration.79/.83 vs..87, NARQ Rivalry.77/.75 vs..83 [97]; Dirty Dozen Machiavellianism.75/.78 vs..76 to.80, Dirty Dozen Psychopathy.43/.67 vs..60 to.72, Dirty Dozen Narcissism.76/.84 vs..74 to.85 [99]).

**Table 13:** CONNECT selected traits and cognitive abilities. Descriptives.

	T1-Self-report			T1-Informant-report^a^			T2-Self-report			T3-Self-report			T4-Self-report			T5-Self-report		
	*M*	*SD*	α	*M*	*SD*	α	*M*	*SD*	α	*M*	*SD*	α	*M*	*SD*	α	*M*	*SD*	α
Age	21.31	3.95		28.51	13.52		21.14	3.60		21.75	3.81		22.36	3.54		23.37	3.42	
Gender	101 ♀	23♂		172 ♀	72♂		85 ♀	21♂		91 ♀	17 ♂		85 ♀	14 ♂		76 ♀	16 ♂	
**Big Five**																		
Neuroticism	4.61	1.30	.77	4.03	1.05	.73	4.38	1.32	.81	4.22	1.36	.84	3.95	1.37	.84	3.96	1.33	.84
Extraversion	5.11	1.11	.85	5.39	0.94	.80	5.15	0.98	.77	5.20	0.94	.77	5.11	1.19	.87	5.10	1.14	.86
Openness	5.15	1.17	.72	5.17	0.87	.67	5.06	1.11	.76	5.08	1.22	.79	5.07	1.25	.83	5.04	1.15	.80
Agreeableness	5.02	0.79	.56	5.34	0.74	.71	4.91	0.76	.57	4.94	0.72	.52	5.13	0.85	.70	5.14	0.86	.74
Conscientiousness	5.45	1.06	.71	6.11	0.80	.76	5.15	1.06	.72	5.05	1.04	.72	5.18	1.08	.75	5.09	1.05	.72
**Self-esteem and self-concept**																		
RSES	3.21	0.56	.89	3.26	0.40	.89	3.19	0.55	.91	3.25	0.47	.86	3.29	0.51	.88	3.33	0.57	.92
SAQ	6.37	0.86	.66	7.26	0.73	.69	6.29	0.86	.68	6.35	0.79	.62	6.32	0.78	.63	6.26	0.76	.64
**Narcissism and dark triad**																		
NPI^b^	14.93	5.76	.78	4.62	2.69	.81	13.09	5.01	.73	13.68	5.26	.74	12.41	5.64	.80	12.04	4.60	.69
NARQ-Admiration	3.17	0.69	.79	3.07	0.64	.83	3.07	0.67	.79	3.22	0.69	.83	3.08	0.77	.85	3.00	0.73	.83
NARQ-Rivalry	2.10	0.61	.77	1.81	0.53	.85	2.01	0.61	.80	2.15	0.64	.81	1.94	0.57	.76	1.88	0.54	.75
DIDO-Machiavellianism	2.78	1.36	.75	2.12	0.90	.75	2.77	1.31	.76	3.09	1.42	.83	2.77	1.24	.71	2.79	1.35	.78
DIDO-Psychopathy	2.38	1.09	.43	2.16	0.97	.67	2.46	1.11	.49	2.75	1.31	.64	2.48	1.22	.66	2.44	1.26	.67
DIDO-Narcissism	4.39	1.57	.76	3.25	1.45	.84	4.13	1.73	.82	4.56	1.56	.82	4.28	1.64	.80	4.10	1.77	.84
**Cognitive abilities** (between T3 and T4)																		
WMC — solved equations	52.15	5.35	.73															
WMC — memory span	5.11	2.33	.71															
MWTB	29.36	1.87	.19															
Short Raven	9.32	2.48	.56															

*Note*. *N*s range from 92 to 124. *M* = mean, *SD* = standard deviation, *α* = Cronbach’s alpha. RSES = Rosenberg Self-Esteem Scale, SAQ = Self-Attributes Questionnaire, NPI = Narcissistic Personality Inventory, NARQ = Narcissistic Admiration and Rivalry Questionnaire, DIDO = Dirty Dozen, WMC = Working memory capacity, MWTB = Vocabulary Knowledge Test [Mehrfachwahl-Wortschatz-Test]. Scales: Big Five 1 to 7, RSES 1 to 4, SAQ 1 to 10, NPI 0 to 40, NARQ 1 to 6, DIDO 1 to 9, WMC — solved equations 0 to 60, WMC — memory span 0 to 8, MWTB 0 to 36, Short Raven 0 to 15.

^a^Informant-results for *M* and *SD* refer to the mean acquaintance report per person. For alpha results, informant-ratings were aggregated before calculation.

^b^A short version (15 items, range 0 to 15) of the NPI was used for the informant-report.

Intercorrelations between the trait variables can be found in Table 14. We found medium to high correlations [125] between self- and informant-reports, which were similar to those found in PILS and in a meta-analysis by Connelly and Ones [30] (our data vs. self-family agreement/self-friend agreement; neuroticism:.60 vs..43/.33, extraversion.56 vs..48/.40, openness:.58 vs..43/.33, agreeableness:.32 vs..37/.29, conscientiousness:.57 vs..42/.38). We found significant correlations between Big Five traits that were similar to meta-analytical results (cf. [126]; our data vs. meta-analysis: extraversion and openness.29 vs..31, agreeableness and conscientiousness.29 vs..31, neuroticism and agreeableness -.18 vs. -.31, extraversion and agreeableness.23 vs..18). As in PILS, there was no significant association between neuroticism and conscientiousness (-.07 our data vs. -.32 meta-analysis). Also, we did not find a significant correlation between neuroticism and extraversion (-.10 our data vs. -.26 meta-analysis).

**Table 14:** CONNECT selected traits and cognitive abilities. Correlations.

	1	2	3	4	5	6	7	8	9	10	11	12	13	14	15	16	17
1 Neuroticism	**.*60***	-.10	-.05	**-.18**	-.07	**-.53**	**-.37**	-.12	.00	**.24**	.**19**	-.12	**.23**	.15	-.05	-.06	**-.28**
2 Extraversion	**-.22**	**.*56***	**.29**	**.23**	.03	.17	**.27**	**.23**	**.19**	-.05	.03	-.07	.09	.05	.04	-.12	.06
3 Openness	-.18	**.21**	**.*58***	.14	.07	**.26**	**.36**	**.28**	**.29**	-.16	-.03	-.03	.03	.03	-.02	.10	.11
4 Agreeableness	**-.29**	**.23**	**.21**	**.*32***	**.29**	**.31**	**.28**	.09	.17	**-.25**	**-.28**	**-.29**	-.10	-.01	-.04	-.08	-.01
5 Conscientiousness	**-.21**	.15	.13	.20	**.*57***	**.33**	**.40**	.12	.16	**-.28**	**-.19**	**-.25**	-.03	-.08	-.18	-.18	-.14
6 RSES	**-.57**	**.37**	**.21**	**.30**	**.39**	**.*64***	**.59**	**.31**	**.29**	**-.35**	**-.24**	-.14	**-.22**	-.15	-.02	-.05	-.02
7 SAQ	**-.40**	**.31**	**.29**	**.22**	**.42**	**.54**	**.*23***	**.39**	**.37**	-.06	-.05	-.01	.03	-.08	-.10	-.19	-.07
8 NPI	-.18	**.23**	.15	-.13	.06	**.33**	**.27**	**.*48***	**.57**	.16	**.19**	**.19**	**.18**	-.05	-.02	-.13	.04
9 NARQ-Admiration	-.12	.11	.19	.07	-.04	**.34**	**.25**	**.65**	**.*37***	**.23**	**.20**	.12	**.31**	-.06	.05	-.11	-.03
10 NARQ-Rivalry	**.34**	-.16	**-.23**	**-.49**	**-.23**	**-.28**	-.19	.19	**.27**	**.*19***	**.57**	**.29**	**.55**	.19	.06	.02	-.07
11 DIDO-Machiavellianism	.20	.04	-.06	**-.27**	-.09	-.16	-.08	.17	**.28**	**.65**	**.*31***	**.45**	**.52**	.09	.15	-.02	.01
12 DIDO-Psychopathy	-.06	-.01	.05	**-.31**	-.12	-.12	.02	**.31**	**.23**	**.44**	**.53**	**.*45***	**.33**	-.17	**.28**	.09	.29
13 DIDO-Narcissism	.16	.08	.00	**-.25**	-.20	-.15	-.16	**.36**	**.38**	**.44**	**.48**	**.37**	**.*30***	.09	.07	.14	.06
14 WMC — solved equations	.03	.15	-.04	.22	.02	.02	.03	-.09	-.02	.05	.12	-.18	.03	-	.09	-.01	-.12
15 WMC — memory span	-.11	.10	.05	-.05	.00	.08	.08	.07	.19	.02	.06	.21	.09	.09	-	-.05	**.30**
16 MWTB	-.01	-.05	.07	-.16	-.17	-.08	**-.37**	-.09	-.07	.03	-.03	.06	-.06	-.01	-.05	-	.02
17 Short Raven	-.21	-.04	-.05	-.14	-.10	-.03	.00	.12	.08	-.04	-.05	.18	.12	-.12	**.30**	.02	-

*Note*. *N*s range from 80 to 124. Above the diagonal: Intercorrelations between self-reported traits (T1). Below the diagonal: Intercorrelations between self-reported traits (T5). Diagonal elements (in italics): correlation between self-reported traits (T1) and informant-reported traits (T1). Cognitive abilities were assessed between T3 and T4. Significant correlations (*p* <.05) in bold.

### Results CONNECT — Time-based assessments

Table 15 shows the results from the first 21 time points of the time-based assessment, divided into three phases (seven diaries each). Due to the nature of this data set (naturally occurring network data), we calculated the perceiver, target, and relationship variance by computing crossed-random-effects models (using the R package lme4: [127]). We found that for most interpersonal perceptions and relationship indicators, the relationship variance was much higher than the perceiver or target variances. Notable exceptions were, for example, the perceptions of intelligence (Phase 1/2/3: 49%/48%/56% perceiver variance) or the perceptions of attractiveness (Phase 1/2/3: 32%/31%/27% target variance). Relative to other impressions, agentic characteristics showed a large amount of target variance (e.g., extraversion Phase 1/2/3: 14%/34%/34%; dominance Phase 1/2/3: 26%/26%/24%). This is in line with previous research that showed a substantial consensus when judging agentic characteristics and leadership, respectively [49,129].

**Table 15:** CONNECT selected time-based assessment results.

	Diary 1 to Diary 7/Phase 1							Diary 8 to Diary 14/Phase 2							Diary 15 to Diary 21/Phase 3						
	Self-perceptions			Other perceptions				Self-perceptions			Other perceptions				Self-perceptions			Other perceptions			
	*M*	*SD wit*.	*SD bet*.	*M*	*Per*. *Var*.	*Tar*.*Var*.	*Rel*. *Var*.	*M*	*SD wit*.	*SD bet*.	*M*	*Per*.*Var*.	*Tar*. *Var*.	*Rel*. *Var*.	*M*	*SD wit*.	*SD bet*.	*M*	*Per*.*Var*.	*Tar*.*Var*.	*Rel*. *Var*.
**Interpersonal perceptions**																					
Knowing				2.21	0.15	0.12	0.82				2.52	0.19	0.15	0.93				2.72	0.25	0.16	1.13
Liking	9.12	0.79	1.64	7.37	0.73	0.22	2.12	9.06	0.54	1.78	7.27	0.70	0.21	2.01	9.03	0.70	1.75	7.21	0.72	0.19	1.96
Metaliking				7.28	0.75	0.11	1.58				7.20	0.73	0.12	1.52				7.14	0.74	0.10	1.49
Extraversion-Introversion	4.16		2.11	4.41	0.82	1.98	3.04	4.00	0.91	1.67	3.92	0.89	1.72	2.45	4.07	0.85	1.60	3.89	1.27	1.78	2.22
Critical-Warmhearted	8.15		2.14	7.66	1.23	0.35	2.35	8.66	1.04	1.53	8.17	1.12	0.44	2.21	8.68	0.98	1.55	8.48	1.35	0.44	2.09
Reliable-Careless	3.61		2.24	4.11	0.98	0.18	1.85	3.76	1.01	1.98	3.66	0.92	0.39	1.78	3.51	0.68	1.69	3.36	0.78	0.88	1.72
Anxious-Stable	7.49		2.32	7.90	1.16	0.45	2.06	7.37	1.02	2.19	8.19	1.09	0.44	1.98	7.58	1.10	2.09	8.33	1.10	0.67	2.22
Interested-Conventional	3.10		1.42	4.36	1.10	0.24	2.22	3.16	0.86	1.25	3.82	1.16	0.34	1.90	3.10	0.74	1.42	3.63	1.19	0.42	1.92
Dominant-Submissive	4.32		1.76	4.33	0.86	1.11	2.25	4.27	0.86	1.52	3.89	0.89	0.92	1.72	4.06	0.76	1.34	3.67	1.14	0.81	1.44
Affectionate-Coldhearted	2.51		1.16	3.81	0.75	0.22	1.92	2.64	0.69	0.93	3.29	0.63	0.22	1.42	2.57	0.71	0.99	3.00	0.79	0.25	1.20
Intelligent-Unintelligent	2.98		1.22	3.57	1.19	0.09	1.15	3.11	0.54	1.07	3.05	0.85	0.13	0.80	3.10	0.44	1.09	2.83	0.98	0.14	0.65
Attractive-Unattractive	4.03		1.85	4.56	1.06	1.43	2.04	3.83	0.70	1.60	4.00	1.02	1.18	1.62	3.68	0.49	1.58	3.87	1.60	1.10	1.40
Admiration	3.59		2.23	3.21	2.01	0.63	2.16	3.42	0.85	1.98	3.09	2.14	0.49	1.96	3.17	0.70	1.92	2.61	2.13	0.40	1.29
Rivalry	2.72		1.96	2.83	2.02	0.31	1.87	2.71	0.77	1.73	2.68	1.94	0.27	1.67	2.57	0.65	1.67	2.39	1.83	0.28	1.41
**Relationship indicators**																					
Satisfaction				7.81	1.00	0.09	2.37				8.35	1.17	0.09	2.03				8.72	0.98	0.17	1.84
Importance				6.91	1.90	0.35	3.98				7.72	1.86	0.29	3.76				8.32	1.56	0.45	3.23
Conflict				1.95	1.44	0.03	0.90				2.07	1.24	0.07	1.19				2.07	0.93	0.17	1.24
Emotional support				5.27	4.20	0.33	3.84				6.05	4.78	0.51	4.10				6.72	3.88	0.89	4.20
Instrumental support				6.78	3.02	0.18	3.53				7.26	3.50	0.27	3.66				7.90	2.73	0.45	3.21
Exchange				6.90	2.42	0.22	3.54				7.55	2.56	0.28	3.17				8.07	2.06	0.36	2.71
Acceptance				8.22	1.44	0.11	2.07				8.68	1.48	0.15	2.00				9.15	1.23	0.12	1.51

*Note*. *N*s range from 108 to 124 for perceivers and from 127 to 131 for targets. *M* = mean aggregated across individual perceiver means, *SD* wit. = standard deviation within perceivers, *SD* bet. = standard deviation between perceivers. *Per*. *Var*. = Perceiver Variance, *Tar*. *Var*. = Target Variance, *Rel*. *Var*. *=* Relationship Variance. Scales: knowing 1 to 6, all other variables 1 to 11.

### Results CONNECT — Event-based assessments

Corresponding to the time-based assessment displayed in Table 15, Table 16 displays the results for the event-based assessment. In total, participants reported on 6,711 interactions, ranging from 2 to 134 interactions per participant (*M* = 54.56, *SD* = 25.80, *Mdn* = 56). The results presented here are split into three phases (Phase 1: Weeks 1 to 3; Phase 2: Week 4; Phase 3: Week 5). We compared our ICCs (percentage of the total variance accounted for by between-person variance) for self-reported behaviors with a recent experience sampling study by Sherman, Rauthmann, Brown, Serfass, and Jones [149]. For behaviors that were assessed in both studies, we found very similar results (our data Phase 1 vs. Sherman et al.: dominance 40% vs. 38%, sociability 42% vs. 29%, friendliness/agreeableness 49% vs. 37%, arrogance/humility 51% vs. 46%). Again, we calculated crossed-random-effects models for social relations analyses. As in the time-based assessment, most variance in perceived behaviors was due to unique perceptions of a specific partner (on average 52% relationship variance). There was also substantial perceiver variance (43%) but much less target variance (6%). Again, the largest target variances were found for dominance (11%) and sociability (7%).

**Table 16:** CONNECT selected event-based assessment results.

	Phase 1							Phase 2							Phase 3						
	Self-perceptions			Other perceptions				Self-perceptions			Other perceptions				Self-perceptions			Other perceptions			
	*M*	*SD wit*.	*SD bet*.	*M*	*Per*. *Var*.	*Tar*.*Var*.	*Rel*. *Var*.	*M*	*SD wit*.	*SD bet*.	*M*	*Per*.*Var*.	*Tar*. *Var*.	*Rel*. *Var*.	*M*	*SD wit*.	*SD bet*.	*M*	*Per*.*Var*.	*Tar*.*Var*.	*Rel*. *Var*.
**Behavior ratings**																					
Dominant-Submissive	3.60	0.69	0.45	3.57	0.24	0.14	0.58	3.48	0.60	0.61	3.45	0.38	0.10	0.50	3.53	0.61	0.56	3.45	0.40	0.07	0.40
Sociable-Reclusive	2.38	0.81	0.57	2.25	0.30	0.10	0.72	2.32	0.74	0.58	2.17	0.31	0.07	0.59	2.27	0.71	0.57	2.12	0.34	0.04	0.43
Friendly-Unfriendly	1.97	0.58	0.58	1.88	0.32	0.04	0.48	1.98	0.62	0.58	1.86	0.33	0.04	0.41	1.92	0.54	0.54	1.81	0.27	0.05	0.35
Arrogant-Modest	5.05	0.74	0.77	5.11	0.60	0.08	0.72	5.19	0.67	0.80	5.26	0.62	0.09	0.53	5.23	0.58	0.84	5.30	0.78	0.06	0.41
Exploiting-Cooperative	5.57	0.72	0.70	5.57	0.48	0.03	0.62	5.67	0.74	0.64	5.70	0.49	0.04	0.49	5.74	0.64	0.68	5.78	0.52	0.04	0.48
Self-revealing-Reserved	2.98	0.99	0.74	3.02	0.43	0.09	1.12	2.88	0.97	0.77	2.85	0.57	0.07	0.90	2.82	0.95	0.78	2.77	0.61	0.07	0.81
Reliable-Unreliable	2.49	0.69	0.84	2.47	0.61	0.03	0.60	2.34	0.63	0.81	2.20	0.52	0.05	0.52	2.16	0.59	0.76	2.11	0.49	0.06	0.42
**State affect**																					
Good mood-Bad mood	2.27	0.79	0.59					2.32	0.77	0.64					2.37	0.75	0.62				
Bored-Activated	5.28	1.08	0.52					5.35	0.94	0.53					5.34	0.92	0.58				
Nervous-Relaxed	5.54	0.87	0.73					5.61	0.78	0.78					5.52	0.86	0.77				
Inhibited-Determined	5.22	0.84	0.68					5.24	0.72	0.73					5.22	0.76	0.84				
Satisfied with myself	5.28	1.08	0.77					5.30	0.87	0.93					5.43	0.83	0.86				
Dissatisfied with interaction	2.26	0.93	0.63					2.13	0.82	0.59					2.09	0.75	0.54				
**Interaction ratings**																					
Interesting-Uninteresting				2.32	0.35	0.04	0.81				2.26	0.48	0.06	0.52				2.23	0.42	0.04	0.44
Positive-Negative				2.06	0.29	0.06	0.72				1.97	0.36	0.05	0.49				1.92	0.32	0.06	0.39
**Interaction meta-ratings**																					
Interesting-Uninteresting				2.51	0.43	0.03	0.72				2.36	0.53	0.04	0.45				2.29	0.46	0.02	0.38
Positive-Negative				2.26	0.38	0.04	0.64				2.10	0.45	0.04	0.40				2.07	0.38	0.03	0.34

*Note*. *N*s range from 90 to 122 for perceivers and 120 to 131 for targets. *M* = mean aggregated across individual perceiver means, *SD* wit. = standard deviation within perceivers, *SD* bet. = standard deviation between perceivers. *Per*. *Var*. = Perceiver Variance, *Tar*. *Var*. = Target Variance, *Rel*. *Var*. *=* Relationship Variance. Phase 1 did not include the first 2 days (i.e., zero-acquaintance experiment and day after). Scales: all variables 1 to 7.

#### Results CONNECT — Direct observations

Finally, all behavior and attractiveness ratings (five to seven raters) can be found in Table 17 (range of ICCs from.34 to.89). The low ICC for arrogance (.34) indicates that there was low consensus in judging arrogance on the basis of the small-talk situation. The correlations between aggregated behavior ratings and corresponding self- and informant-reported traits (T1) were similar to PILS results (e.g., self-reported/informant-reported: nervousness and trait neuroticism.09/.11; dominance-expressiveness and trait extraversion.37/.40; friendliness-warmth and trait agreeableness.10/.07; arrogance and average trait Dirty Dozen.21/.27; arrogance and trait narcissistic rivalry.08/.18, intelligent behavior and trait Raven score.19, also see [109], for similar procedures).

**Table 17:** CONNECT selected behavior and attractiveness ratings.

Tasks and variables	*M*	*SD*	*ICC*
**Attractiveness (zero-acquaintance)**			
Attractiveness face	4.58	1.37	.83
Hardness of face	4.41	1.04	.65
Styled hair	4.95	1.35	.78
Neat hairstyle	6.58	1.04	.70
Flashy clothes	4.13	1.27	.85
Neat clothes	6.28	0.81	.70
Modern clothes	4.68	1.39	.81
Attractiveness body	5.16	1.31	.79
**Attractiveness (laboratory)**			
Attractiveness face	4.93	1.44	.81
Hardness of face	4.51	1.14	.69
Styled hair	4.69	1.48	.82
Neat hairstyle	6.50	1.09	.69
Flashy clothes	4.22	1.48	.89
Neat clothes	6.65	0.82	.72
Modern clothes	4.55	1.28	.71
Attractiveness body	5.33	1.31	.78
**Self-introduction (zero-acquaintance)**			
Expressiveness	2.96	0.88	.81
Self-confidence	3.40	0.86	.78
Arrogance	1.55	0.46	.55
Friendliness	3.53	0.71	.78
Nervousness	2.46	0.84	.78
**Small talk (laboratory)**			
Expressiveness	2.97	0.95	.89
Arrogance	1.79	0.52	.34
Warmth	3.92	0.73	.86
Nervousness	1.95	0.52	.59
**Self-introduction (laboratory)**			
Expressiveness	2.84	0.81	.86
Self-confidence	3.16	0.79	.79
Arrogance	1.73	0.68	.58
Warmth	3.84	0.65	.66
Nervousness	2.39	0.64	.68
**Stress test (laboratory)**			
Expressiveness	2.58	0.78	.85
Self-confidence	3.42	0.72	.79
Nervousness	2.57	0.64	.65
Intellectual competence	3.75	0.65	.79
**Public Goods Game (laboratory)**			
1 (money given to small group)	4.30	1.05	
2 (money given to all participants)	3.89	1.51	

*Note*. *N*s range from 88 to 126. *M =* mean rating of participants. *SD* = standard deviation between participants. ICC = intraclass correlation (2,k). Scales: attractiveness ratings 1 to 10, behavior ratings 1 to 6, Public Goods Game 0€ to 5€. Behavior and photographs were rated by five to seven independent raters.

## General discussion

In developmental, social, and personality psychology, researchers aim at opening the process black box that underlies the expression, development, and mutual influence of personality and social relationships. Building on an existing process framework for personality and social relationships (e.g., PERSOC), we deduced four methodological challenges involved in this endeavor. Designed to address these challenges, we presented detailed descriptions of a laboratory-based (PILS) and a field-based (CONNECT) multimethodological, longitudinal, and process-oriented study, respectively. To provide an idea of the data obtained with these projects, the presentation also involved descriptive results. Both, PILS and the CONNECT data sets, focused on student peer-relations. That is, they provide access to relevant behavioral and perceptual processes that underlie the reciprocal interplay of students’ personality and peer relationships in young adulthood in transition to or at University.

### Methodological solutions: Combining laboratory and field

We described four methodological challenges that occur when studying the processes that underlie the reciprocal relation between personality and social relationships. Our complementary laboratory-based and field-based approaches aimed at taking up on these challenges, with each providing unique benefits and strengths (see [68], for an overview).

In the field-based approach in CONNECT, but not in the laboratory-based approach in PILS, we aimed at capturing personality development by repeatedly assessing personality over the course of three years. To be able to detect potentially quick changes directly after the participants transitioned into student life, we realized the first two assessments only being three months apart. Three more waves followed 9, 21, and 33 months after the first assessment to capture comparatively slower changes over the course of participants’ undergraduate studies.

To target the second aim of capturing social relationships, we longitudinally assessed objective and subjective indicators of social relationships, ensuring zero-acquaintance to be the starting point of our investigations in both studies. We captured initial phases of the acquaintance process with more frequent assessments in both studies. To account for the bidirectional nature of social relationships, we employed round-robin designs within small groups in PILS and within the whole cohort of psychology freshmen in CONNECT that captured actor and partner perspectives within dyads and investigated all participants in multiple dyads. Compared to PILS, we enlarged the temporal resolution in CONNECT from hours to weeks, months, and years with regular assessments at later stages when social relationships were already formed, maintained, or terminated.

To realize the third aim of capturing social situations, we sampled social situations in the acquaintance process either in a controlled laboratory approach that resembled the natural acquaintance process or in a naturalistic field approach from participants’ consequential real lives. In PILS, we carefully created social situations to psychologically resemble the natural process of getting to know strangers. Typically increasing in intimacy and interactions depth, the sequence of interactive tasks mirrored this developmental course. Due to the field-based, naturalistic nature of CONNECT, we–somewhat automatically–obtained a representative and typical sample of social situations and their affordances within a new and consequential context of participants’ lives. The event-based assessment in particular was designed to assess rather objective and categorical (i.e., selecting a situation from a list of 17 options) as well as subjective and dimensional situation information (i.e., positive, interesting).

To address the fourth aim of capturing interaction processes, we realized longitudinal and multimethodological designs to repeatedly obtain direct observations (video-based behavioral observations) and immediate reports, capturing objective as well as subjective perspectives. Applying round-robin designs, each participant of the studies was investigated both as actor and partner within multiple dyads. Especially the time- and event-based assessments within CONNECT are unique in the sense that they used the relatively young experience-sampling approach not only to target self-perceptions but also to target other-perceptions.

By combining two state-of-the-art approaches and their advantages, we aimed at providing a realistic portrayal of the expression, development, and interplay of students’ personality and their peer relationships in young adulthood and the underlying processes in question. To simplify the combination of both approaches, we additionally aimed at parallelizing the approaches as much as possible with respect to (a) the participants and the relationship type, (b) the procedures, and (c) the selection of constructs and measures. In both studies, we targeted students and their peer relationships because (the transition to) student life represents a decisive developmental phase in young adulthood and peer relations reflect an important type of social relationships during that time. In both studies, we also started the assessment at the very beginning of the emergence of relationships (i.e., at zero acquaintance) and intensively captured the initial period of acquaintance processes with fine-grained and multimethodological designs. Finally, in both studies, we parallelized the selection of targeted constructs and measures wherever possible. Regarding all construct domains, we applied identical or similar measures that were suited for either the laboratory-based or the field-based settings. In this way, one is able to address the same set of research questions with both data sets, allowing for stronger inferences about the social interaction processes that drive the expression, development, and mutual influence of personality and social relationships in that life phase.

We hope that future research will combine the two methodological approaches forwarded here, to comprehensively investigate the link between personality and social relationships. The combination of laboratory- and field-based designs might help to provide more robust and method-independent insights into this dynamic interplay. The described methodological set-ups can be transferred to and extended in other transitional contexts (e.g., transitions into romantic relations and marriage, into phases of unemployment, and into retirement) and other types of social relationships in other social contexts (e.g., romantic relationships, family relationships, social relationships at the workplace). All these contexts allow for longitudinal assessments of personality and social relationships, with the opportunity of covering accelerated initial and slower long-term changes that should be targeted simultaneously in future studies. With that being said, the field-based approach targeting behavior and social situations could further be augmented with novel methodological solutions regarding the direct assessment of behavior and interpersonal perceptions as life unfolds. For example, the EAR [150,151] intermittently registers snippets of ambient sounds that can be used to infer the state behaviors participants engage in (e.g., laughing, helping, arguing) and affective responses (e.g., being happy, being angry, being sad). Moreover, rapid technical progress further facilitates repeated assessments through new experience sampling tools [152–154] (see [68], for an overview), through wearables (e.g., narrative clip; getnarrative.com) and augmented and virtual reality. The design of future studies could incorporate these novel opportunities of obtaining realistic and meaningful information on behavioral and interpersonal processes within interaction units.

### Opening the process black box: Targeted research domains and exemplary research questions

PILS and CONNECT, and future studies applying similar and extended approaches, offer a variety of opportunities within a process-perspective on the expression, development, and mutual influence of personality and social relationships. First, they allow to capture the expression of personality and social relationships in social action. Although the strong need for more truly behavioral data has been put forward by many scholars in personality and social psychology [3,7,155–157], there are still very few insights into how certain personality characteristics and relationship qualities translate into behavioral and mental differences and into differences in their contingencies during real-life social interactions (e.g., How do narcissistic or conscientious individuals feel, behave, and perceive during different kinds of social interactions? By means of which individual and dyadic behavioral, affective, and perceptual states can more or less good friends, more or less satisfying relationships, be distinguished? e.g.,[158–163]). Such a process-oriented approach to the expression of personality and social relationships might also help researchers to obtain a better understanding of the structure and interrelations of traits and relationship qualities. For example, personality dimensions might be correlated to the extent that they share certain process units (e.g., narcissism and extraversion are both characterized by self-assuredness, dominant behaviors, and optimistic meta-perceptions; see [1]; also see [164], for a related approach), which, in turn, might enable important conclusions regarding the definition, structure, and understanding of personality and in an analogous way, of social relationships.

Second, PILS and CONNECT might be applied to gain a microlevel understanding of how individual personality traits and social relationship qualities develop over time (e.g., What are the behavioral and mental social interaction processes through which individual and relationship characteristics become stable or change? Are there specific life events that drive these changes? And what are the behaviors, affective states, and interpersonal perceptions by which these experiences get under the skin?; e.g., [58,165,166]) Do such potential changes gradually evolve (e.g., based on certain gradually changing behavioral and mental state dynamics) or do they quickly become apparent given specific events (e.g., characterized by specific novel behavioral or mental states; e.g., [53,167])? Based on intensive and longitudinal approaches that capture the expressions of personality and social relationships at multiple time points and perhaps more intensively during the initial phases of relationship emergence, stabilization, and change can be modeled, and the behavioral and interpersonal processes underlying these dynamics can be unraveled (e.g., [168,169]).

Third, both studies align with a process-based understanding of the mutual influence of personality and social relationships. An increasing number of studies has shown that personality shapes the development of social relationships (see [1,7], for an overview). PILS and CONNECT showcase a way to move forward and take a close look at the developing behavioral and mental interaction processes through which the social power of personality can be explained (e.g., Why are narcissists popular at zero acquaintance but less popular later on? Are neurotic individuals particularly unhappy when surrounded by disagreeable social partners—and if yes, why? Through which processes do actual or perceived personality similarity lead to unique liking and friendship?). Similarly, such data can be used to unravel the social interaction state processes that underlie the effect of social relationship experiences on the development of personality (e.g., How exactly does relationship closeness or the size of one’s social network affect the development of self-esteem? What are the shared behavioral processes through which peers within social groups co-develop in their level of conscientiousness? Which (contingencies of) behaviors, affective states, and perceptions need to be triggered so that a new close relationship is effective at strengthening individuals’ emotional stability? Do differences in these event-contingent experiences explain interindividual differences in intraindividual personality change? e.g., [170–174]).

In addition, these studies also illustrate that even more specific domains of research questions can be addressed, for example, those that investigate the description, determinants, and consequences of social interaction dynamics themselves (e.g., How much are social behaviors and interpersonal perceptions related within and between individuals, and how much do individuals and dyads differ herein? Which personal and relational factors influence these differences, and what kinds of intra- and interpersonal consequences do they have?). With regard to the relation between social interaction states within individuals, longitudinal state data, such as those obtained in PILS and CONNECT, can, for example, be applied to investigate in detail interindividual differences in intraindividual behavioral, affective, and perceptual variability versus consistency (see, e.g., [175–177]) as well as individual differences in the amount of authenticity (i.e., consistency between self-perceptions and behavioral expressions). With regard to the relation between social interaction states across social partners, both studies exemplify the opportunity of fine-grained investigations of individual differences in self-other agreement in trait- and behavioral perceptions, the meta-accuracy of interpersonal perceptions, as well as the reciprocity of behaviors, affect, and interpersonal perceptions.

Moreover, complex longitudinal, multi-methodological, multi-perspective (e.g., dyadic, group, and network) data provide a strong basis for developing and evaluating novel statistical techniques particularly regarding multivariate longitudinal analyses. Most of the outlined research domains require the simultaneous longitudinal modeling of personality, relationship, and state dynamics. In addition, they demand a variety of complex mediation and moderation analyses, thereby allowing for different time frames, missing data, and nested data structures. Whereas a number of sophisticated analytic tools have already been developed (see [78,82], for a recent overviews), a variety of novel solutions are needed. Such complex data provide rich opportunities for researchers interested in the development of novel and complex statistical solutions, for example, in the domains of (cross-classified) structural equation modeling, multilevel modeling, continuous time modeling, or multivariate, longitudinal response surface, social network, and social relations analyses (e.g., [78,178–180]).

On a final note, although PILS and CONNECT illustrate how to open the process black box to explain the expression, development, and mutual influence of personality and social relationships, it is important to note that both data sets are not without limitations. Most obviously, in order to provide the basis for concrete research investigations, we were not able to capture personality and social relationships in general but had to narrow down our focus considerably. Specifically, we focused on a specific population (i.e., students), a specific relationship type (i.e., peer relationships), and a specific life phase (i.e., transition into student life), leaving questions of generalizability to other populations, relationship types, and life phases open. In addition, we would like to point out that, at the time of data collection, theoretical and empirical knowledge on the nature of relevant processes was even weaker than today. Therefore, not all of the design-related decisions (e.g., decisions on the timing of assessments, on which variables and constructs to assess and from which perspective) were strongly determined by theoretical considerations or previous empirical work. Instead, they were often based on available indirect scientific knowledge and on informed plausibility assumptions for the studies’ context of students’ peer relationships at university. Hence, in closing this section, we would like to emphasize that PILS and CONNECT constitute starting points, which hopefully paves the way for more–theoretically and empirically grounded–future process-based research across samples, relationship types, and life phases.

### Open research policy: An invitation to collaborate

Trying to empirically analyze the social interaction processes that explain the expression, development, and mutual influence of personality and social relationships, as attempted within PILS and CONNECT, requires the longitudinal collection of a variety of different kinds of data using different specialized techniques as well as rigorous data preparation and complex statistical analyses. In our view, the best way to address these challenges is to join forces with respect to data, resources, and expertise [7]. By combining rich data sets from different laboratories across the world and collaboratively analyzing these data sets, the research field will be able to move on much faster and generate more powerful, thoughtful, and replicable insights. Importantly, pooling smaller but rich data sets will allow to move beyond the trade-off between large and representative samples of participants and rich, psychologically representative process data (e.g., [181]).

In this spirit, our open research policy involves (a) open material, (b) open code, and (c) open data. We provide open material with detailed descriptions of PILS and CONNECT in the Open Science Framework (PILS: osf.io/q5zwp [85]; CONNECT: osf.io/2pmcr [86]). By providing open code in our publications on PILS and CONNECT data, we have already made (i.e., [78,158–161,163,168–176,179,180,182]) and will continue to make public all analytical codes needed to comprehend and reproduce the results presented in our articles (e.g., R codes, Mplus codes; see osf.io/5tw8b/ for this paper). Herewith, we also provide open data for the all descriptive and exemplary analyses (also see osf.io/zj38h/) to actually offer other researchers the opportunity to reproduce the published results with the provided analytical code. The *full* anonymized data sets of these two projects that is, data sets also including those variables that were not the basis for the descriptive, reliability, and intercorrelation information as well as the exemplary analyses presented in the main text and the supplement of this manuscript will be made publicly available in the OSF on January 01, 2021. Before that date, we cordially invite researchers to use these data and are more than happy to collaborate. The best and easiest way to obtain the data is via a structured request (forms available at PILS: osf.io/q5zwp [85]; CONNECT: osf.io/2pmcr [86]). We hope that this open policy will encourage fruitful research, discussions, and collaborations on the dynamic interplay of personality and social relationships.

## Conclusion

With the present paper, we aimed to contribute to a better understanding of the processes underlying the expression, development, and mutual influence of personality and social relationships. We deduced four methodological challenges and presented two longitudinal, and multimethodological data sets designed to address these challenges in a laboratory-based and a field-based context: The PILS and the CONNECT Study. We hope that these projects will help to promote collaborative research efforts focused on understanding the interplay of students’ personality and their peer relationships and will stimulate related research efforts across other populations, relationship types, and life phases.

## Supporting information

S1 Table Sources and domains of multimethodological assessments in PILS and CONNECT. (DOCX)

S2 Table Overview of assessed variables in PILS and CONNECT. (DOCX)

S3 Table Overview of assessed life events in CONNECT. (DOCX)
