# Supplementary material for: Explaining the longitudinal interplay of personality and social relationships in the laboratory and in the field: The PILS and the CONNECT study
Source: PLoS One. 2019 Jan 30;14(1):e0210424. doi: 10.1371/journal.pone.0210424 (PMC6353144; doi:10.1371/journal.pone.0210424)
Supplement: S1 Table — (DOCX) [file pone.0210424.s001.docx]

**Supporting Information 1**

**S1 Table. Sources and domains of multimethodological assessments in PILS and CONNECT**

|  | PILS | | CONNECT | | | | |
| --- | --- | --- | --- | --- | --- | --- | --- |
|  | Online Survey | Session  Data | Zero  Acquaintance | Online Survey  T1-5 | Time-based Assessment | Event-based Assessment | Laboratory Experiment |
| Expressiveness | Extraversion, extraversion other sex, sociability, shyness, shyness other sex, sociability (SR, IR) | Expressiveness (OR) | Expressiveness (OR) | Extraversion, extraversion other sex, sociability, shyness, shyness other sex, sociability (SR, IR), retrospective extraversion (SR) | Extraverted (SR, IP) | Sociable (SR, IP) | Expressiveness (OR), IAT: extraversion (SR) |
| Dominance/ Self-confidence | Self-esteem, self-concept: leadership ability (SR, IR) | Leadership (SR, IP), dominance (OR) | Dominance (IP), self-confidence (OR) | Self-esteem, self-concept: leadership ability, dominance (SR, IR), retrospective dominance (SR) | Dominant (SR, IP), leader (IP) | Dominant (SR, IP) | Self-confidence (OR), IAT: self-esteem (SR), |
| Arrogance | Narcissism: rivalry (SR, IR) | Rivalry (SR, IP) arrogance (OR) | Arrogance (OR) | Narcissism: rivalry, self-concept: arrogance (SR, IR) | Rivalry (SR, IP) | Arrogant (SR, IP) | arrogance (OR) |
| Aggressiveness | Anger, impulsiveness | Aggressiveness (OR) |  | Anger, impulsiveness (SR, IR), retrospective aggressiveness (SR) |  |  |  |
| Friendliness/  Warmth | Trait: agreeableness, self-concept: helpful, sensitive, trustworthy | Trustworthiness, friendliness, cooperativeness (OR) | Affectionate (IP), Friendliness (OR) | Agreeableness, self-concept: helpful, sensitive, trustworthy, affectionate (SR, IR), retrospective warmth & agreeableness (SR) | Critical, affectionate (SR, IP) | Friendly, cooperative (SR, IP), | Friendliness, warmth (OR), trait: social value orientation (SR), IAT: agreeableness (SR) |
| Negative Affect /Nervousness | Neuroticism, need to belong, affect, self-concept: emotional stability (SR, IR) | Nervousness (OR) | Nervousness (SR, OR) | Neuroticism, need to belong, affect, self-concept: emotional stability, insecure (SR, IR), retrospective (neuroticism) (SR) | Anxious (SR, IP) | Nervousness (SR) | Nervousness (OR), IAT: neuroticism (SR) |
| Intelligence | Self-concept: intellectual abilities (SR, IR) | Intelligence (SR, IP, OR), attention, verbal fluency (OR), cognitive abilities: working memory, vocabulary knowledge, reasoning (SR) |  | Self-concept: intellectual abilities (SR, IR), grades: university & high school (SR) | Intelligent (SR, IP) |  | Intellectual (OR), cognitive abilities: working memory, vocabulary knowledge, reasoning (SR) |
| Attractiveness/ Attraction | Self-concept: attractiveness, narcissism: admiration, sociosexual orientation (SR, IR) | Admiration, attractiveness, physical appeal, date, short love affair, romantic relationship (SR, IP), attractiveness (OR) | Attractiveness (OR) | Self-concept: attractiveness, narcissism: admiration, sociosexual orientation (SR, IR) | attractive, admiration (SR, IP), date (IP) |  | Attractiveness (OR) |

*Note.* SR = self-report, IR = informant-report, IP = interpersonal perception, OR = observer rating (based on video or photograph).

Table S2

*Overview of assessed variables in PILS and CONNECT*

|  | PILS | | CONNECT | | | | |
| --- | --- | --- | --- | --- | --- | --- | --- |
|  | Online Survey | Session  Data | Zero  Acqu. | Online Survey  T1-5 | Time-based | Event-based | Lab |
| **Demographics** |  |  |  |  |  |  |  |
| Age | SR |  |  | T1-5 SR |  |  |  |
| Sex | SR |  |  | T1-5 SR |  |  |  |
| Marital status | SR |  |  | T1-5 SR |  |  |  |
| First language | SR |  |  | T1 SR |  |  |  |
| Study situation | SR |  |  | T4-5 SR |  |  |  |
| Height |  |  |  | T5 SR |  |  |  |
| Weight |  |  |  | T5 SR |  |  |  |
| Handedness |  |  |  | T5 SR |  |  |  |
| Sleeping habits |  |  |  | T5 SR |  |  |  |
| Sick days |  |  |  | T5 SR |  |  |  |
| Living situation |  |  |  | T3-5 SR |  |  |  |
| Favorite subjects |  |  |  | T5 SR |  |  |  |
| Romantic relationships |  |  |  | T5 SR |  |  |  |
| Going out frequencies |  |  |  | T5 SR |  |  |  |
| Life satisfaction |  |  |  | T5 SR |  |  |  |
| Staying abroad |  |  |  | T5 SR |  |  |  |
| Visited countries |  |  |  | T5 SR |  |  |  |
| Foreign languages |  |  |  | T5 SR |  |  |  |
| Smoking |  |  |  | T5 SR |  |  |  |
| Alcohol consumption |  |  |  | T5 SR |  |  |  |
| **Trait measures** |  |  |  |  |  |  |  |
| Big Five |  |  |  |  |  |  |  |
| - Neuroticism | SR, IR |  |  | T1-5 SR, T1 IR |  |  |  |
| - Extraversion | SR, IR |  |  | T1-5 SR, T1 IR |  |  |  |
| - Openness | SR, IR |  |  | T1-5 SR, T1 IR |  |  |  |
| - Conscientiousness | SR, IR |  |  | T1-5 SR, T1 IR |  |  |  |
| - Agreeableness | SR, IR |  |  | T1-5 SR, T1 IR |  |  |  |
| Shyness | SR, IR |  |  | T1-5 SR, T1 IR |  |  |  |
| Shyness toward other sex | SR, IR |  |  | T1-5 SR, T1 IR |  |  |  |
| Sociability | SR, IR |  |  | T1-5 SR, T1 IR |  |  |  |
| Extraversion toward other sex | SR, IR |  |  | T1-5 SR, T1 IR |  |  |  |
| Narcissism (NPI-40) | SR |  |  | T1-5 SR |  |  |  |
| Narcissism (NPI-15) | IR |  |  | T1 IR |  |  |  |
| Trait affect and self-esteem | SR, IR |  |  | T1-5 SR, T1 IR |  |  |  |
| Trait Anger | SR, IR |  |  | T1-5 SR, T1 IR |  |  |  |
| Impulsivity | SR, IR |  |  | T1-5 SR, T1 IR |  |  |  |
| Sensation seeking | SR, IR |  |  | T1-5 SR, T1 IR |  |  |  |
| NARQ: Narcissistic admiration | SR, IR |  |  | T1-5 SR, T1 IR |  |  |  |
| NARQ: Narcissistic rivalry | SR, IR |  |  | T1-5 SR, T1 IR |  |  |  |
| Dirty Dozen: Narcissism | SR, IR |  |  | T1-5 SR, T1 IR |  |  |  |
| Dirty Dozen: Machiavellianism | SR, IR |  |  | T1-5 SR, T1 IR |  |  |  |
| Dirty Dozen: Psychopathy | SR, IR |  |  | T1-5 SR, T1 IR |  |  |  |
| Self-concept |  |  |  |  |  |  |  |
| - Intellectual ability | SR, IR |  |  | T1-5 SR, T1 IR |  |  |  |
| - Social skills | SR, IR |  |  | T1-5 SR, T1 IR |  |  |  |
| - Artistic/ musical ability | SR, IR |  |  | T1-5 SR, T1 IR |  |  |  |
| - Athletic ability | SR, IR |  |  | T1-5 SR, T1 IR |  |  |  |
| - Leadership ability | SR, IR |  |  | T1-5 SR, T1 IR |  |  |  |
| - Common sense | SR, IR |  |  | T1-5 SR, T1 IR |  |  |  |
| - Emotional stability | SR, IR |  |  | T1-5 SR, T1 IR |  |  |  |
| - Sense of humor | SR, IR |  |  | T1-5 SR, T1 IR |  |  |  |
| - Discipline | SR, IR |  |  | T1-5 SR, T1 IR |  |  |  |
| - Attractiveness | SR, IR |  |  | T1-5 SR, T1 IR |  |  |  |
| - Specific intellectual abilities | SR, IR |  |  | T1-5 SR, T1 IR |  |  |  |
| - Agentic traits | SR, IR |  |  | T1-5 SR, T1 IR |  |  |  |
| - Communal traits | SR, IR |  |  | T1-5 SR, T1 IR |  |  |  |
| - Antagonistic Traits |  |  |  | T1-5 SR, T1 IR |  |  |  |
| Communal narcissism | SR, IR |  |  | T1-5 SR, T1 IR |  |  |  |
| Self-esteem | SR, IR |  |  | T1-5 SR, T1 IR |  |  |  |
| Need to belong | SR, IR |  |  | T1-5 SR, T1 IR |  |  |  |
| Sociosexual orientations | SR, IR |  |  | T1-5 SR, T1 IR |  |  |  |
| Sexual orientation | SR, IR |  |  | T1-5 SR, T1 IR |  |  |  |
| Implicit personality |  |  |  |  |  |  | SR |
| Social value orientation |  |  |  |  |  |  | SR |
| Cognitive abilities |  |  |  |  |  |  |  |
| - Working memory |  | S1 SR |  |  |  |  | SR |
| - Vocabulary knowledge |  | S1 SR |  |  |  |  | SR |
| - Reasoning |  | S1 SR |  |  |  |  | SR |
| Public Goods Game |  |  |  |  |  |  | SR |
| **Relationship indicators** |  |  |  |  |  |  |  |
| Acquaintance |  | S1 IP | IP |  | A IP |  |  |
| Friendship |  | S1-3 SR, IP |  | T3-T5 | C IP |  |  |
| Leadership |  | S1-3 SR, IP |  |  | C IP |  |  |
| Dating and mating potential |  | S3 SR, IP |  |  | C IP |  |  |
| Interaction frequency |  |  |  | T3-5 SR | B-C IP |  |  |
| Relationship quality |  |  |  | T2-T5 SR | C IP |  |  |
| Broader social network |  |  |  | T1-5 SR | A IP |  |  |
| Social interaction ratings |  |  |  | T3-5 SR |  | SR IP |  |
| **Interpersonal perceptions** |  |  |  |  |  |  |  |
| Liking |  | S1-3 SR, IP | IP |  | A SR, IP |  |  |
| Metaliking |  | S1-3 SR, IP | IP | T3-5 SR | A SR, IP |  |  |
| Annoying |  | S1-3 SR, IP |  |  |  |  |  |
| Attractiveness |  | S1-3 SR, IP |  |  |  |  |  |
| Personality impressions |  | S1-3 SR, IP | IP |  | B SR, IP |  |  |
| Status perceptions |  |  |  | T4-5 SR IP | C SR IP |  |  |
| Retrospective accuracy perceptions | | T3 SR |  | T3-5 SR |  |  |  |
| **Personality state ratings** |  |  |  |  |  |  |  |
| State affect |  | S1-3 SR | SR |  |  | SR |  |
| Affect grid |  | S1-3 SR |  |  |  |  |  |
| State self-esteem |  | S1-3 SR | SR |  |  | SR |  |
| **Physical and behavioral measures** | |  |  |  |  |  |  |
| Physical ratings |  |  |  |  |  |  |  |
| - Face |  | OR | OR |  |  |  | OR |
| - Body |  | OR | OR |  |  |  | OR |
| - Clothes |  | OR | OR |  |  |  | OR |
| - Hair |  | OR | OR |  |  |  | OR |
| Individual behavioral ratings |  |  |  |  |  |  |  |
| - Attention (Voice) |  | S1 OR |  |  |  |  |  |
| - Cheerfulness of voice |  | S1 OR |  |  |  |  |  |
| - Intelligence (Voice) |  | S1 OR |  |  |  |  |  |
| - Verbal fluency (Voice) |  | S1 OR |  |  |  |  |  |
| - Nervousness (Voice) |  | S1 OR |  |  |  |  |  |
| - Arrogant behavior |  | S1-3 OR | OR |  |  |  | OR |
| - Dominant behavior |  | S1-3 OR | OR, IP |  |  |  |  |
| - Expressive behavior |  | S1-3 OR | OR |  |  |  | OR |
| - Friendly behavior |  | S1 OR | OR |  |  |  |  |
| - Nervous behavior |  | S1 OR | OR |  |  |  | OR |
| - Aggressive behavior |  | S2-3 OR |  |  |  |  |  |
| - Cooperative behavior |  | S2-3 OR |  |  |  |  |  |
| - Warm-heartedness |  |  |  |  |  |  | OR |
| - Self-confidence |  |  |  |  |  |  | OR |
| - Intellectual behavior |  |  |  |  |  |  | OR |
| - Affectionate behavior |  |  | IP |  |  |  |  |
| Group behavioral ratings |  |  |  |  |  |  |  |
| - Performance |  | S2-3 OR |  |  |  |  |  |
| - Frequency of conflicts |  | S2-3 OR |  |  |  |  |  |
| - Positive atmosphere |  | S2-3 OR |  |  |  |  |  |
| Interactional behavioral ratings |  |  |  |  |  |  |  |
| - Dominant vs. submissive | |  |  |  |  | SR, IP |  |
| - Sociable vs. reclusive | |  |  |  |  | SR, IP |  |
| - Friendly vs. unfriendly | |  |  |  |  | SR, IP |  |
| - Arrogant vs. modest | |  |  |  |  | SR, IP |  |
| - Exploiting vs. cooperative | |  |  |  |  | SR, IP |  |
| - Self-revealing vs. reserved | |  |  |  |  | SR, IP |  |
| - Reliable vs. unreliable | |  |  |  |  | SR, IP |  |
| **Additional life events & Outcomes** | |  |  |  |  |  |  |
| Social network use | SR |  |  | T1-5 SR |  |  |  |
| Academic achievements |  |  |  | T3-5 SR |  |  |  |
| Life events |  |  |  |  |  |  |  |
| - Family |  |  |  | T5 SR |  |  |  |
| - Friends |  |  |  | T5 SR |  |  |  |
| - Romantic relationships |  |  |  | T5 SR |  |  |  |
| - Personal life |  |  |  | T5 SR |  |  |  |
| - Work |  |  |  | T5 SR |  |  |  |
| - Studies |  |  |  | T5 SR |  |  |  |
| Self-perceived personality development | |  |  | T3-5 SR |  |  |  |
| *Note. Zero Acqu. =* zero-acquaintance experiment. SR = self-report, IR = informant-report, IP = interpersonal perception, OR = observer rating, S = sessions 1 to 3, T = time points 1 to 5. | | | | | | | |

Table S3

*Overview of assessed life events in CONNECT*

| Family | Family member got seriously ill or badly injured | | |
| --- | --- | --- | --- |
|  | Family member passed away | | |
|  | Parents split up or got divorced | | |
|  | Closeness to family members changed | | |
|  | Family gained a new family member | | |
| Friends | Close friend got seriously ill or badly injured | |  |
|  | Close friend passed away | | |
|  | Made a new close friend | | |
|  | Serious conflict with a close friend | | |
|  | Gave up a close friendship | | |
| Romantic Relationships | Started being in a relationship |  |  |
|  | Partner got badly injured or was seriously ill | | |
|  | Partner passed away | | |
|  | Serious relationship crisis | | |
|  | Number of conflicts with partner changed | | |
|  | Problems with your partner’s family | | |
|  | Partner problems with participant’s family | | |
|  | Romantic relationship to former partner ended | | |
|  | Made up after a serious relationship crisis | | |
|  | Engaged | | |
|  | Married | | |
|  | Divorced | | |
|  | Spatial proximity or distance to partner changed | | |
|  | Partner’s work habits significantly changed | | |
|  | Participants’/partners’ pregnancy | | |
|  | Participants’/partners’ abortion | | |
|  | Sexual problems | | |
| Personal Life | Got badly injured or was seriously ill | | |
|  | Personal meaningful events | | |
|  | Spent at least 1 month at a stretch abroad for no professional or education-related reasons (private) | | |
|  | Spent at least 1 month abroad, for example, due to an internship or your studies | | |
|  | Time spend on social activities (e.g., parties, cinema, cultural activities, cooking together) changed | | |
|  | Type of social activities changed | | |
|  | Changed sleeping habits | | |
|  | Changed quantitative eating habits | | |
|  | Changed qualitative eating habits | | |
|  | Changed alcohol consumption habits | | |
|  | Changed smoking habits | | |
|  | Moved out from parental home | | |
|  | Moved (apart from moving out from parental home) | | |
|  | If lived in a shared flat: serious problems in shared flat | | |
|  | Financial situation significantly changed | | |
|  | Problems with living expenses or concerned about not being able to finance the continuation of studies | | |
|  | Borrowed a major amount of money (more than 1,000 Euro) from someone (apart from BAfÖG or something similar) | | |
|  | Went through a compulsory execution or pledging of goods | | |
|  | Got caught because of a minor breaking of law (e.g., fare dodging, speeding, etc.) | | |
|  | Been in jail or a comparable institution (e.g., a drunk tank, a psychiatric clinic, or something similar) | | |
|  | Went to or started psychotherapy | | |
|  | Failed an important nonuniversity exam (e.g., driving test) | | |
|  | Important nonuniversity exam (e.g., driving test) not attended | | |
| Work | Worked before starting studies or worked part-time while studying or accepted a new job | | |
|  | Working hours at job increased significantly | | |
|  | Promoted or responsibility at job significantly increased | | |
|  | Conflicts with employer which brought up the risk of losing job, being suspended or being demoted to a lower rank | | |
|  | Quit job (by participant or employer) | | |
| Studies | Dropped out or changed course of studies or apprenticeship | | |
|  | Graduated from high school | | |
|  | Failed an university exam | | |
|  | Did not attend an important university exam | | |
|  | Doubts about continuing studies | | |
|  | Transferred to another university | | |
|  | Started another academic course (at university, technical college, or training school) above studies of psychology | | |
|  | Graduated from an academic course other than psychology course | | |
| Other | Any further incident that had an impact on life | | |
