# Supplementary material for: Explaining the longitudinal interplay of personality and social relationships in the laboratory and in the field: The PILS and the CONNECT study
Source: PLoS One. 2019 Jan 30;14(1):e0210424. doi: 10.1371/journal.pone.0210424 (PMC6353144; doi:10.1371/journal.pone.0210424)
Supplement: S2 Table — (DOCX) [file pone.0210424.s002.docx]

**Supporting Information 2**

**Table S2. Overview of assessed variables in PILS and CONNECT**

|  | PILS | | CONNECT | | | | |
| --- | --- | --- | --- | --- | --- | --- | --- |
|  | Online Survey | Session  Data | Zero  Acqu. | Online Survey  T1-5 | Time-based | Event-based | Lab |
| **Demographics** |  |  |  |  |  |  |  |
| Age | SR |  |  | T1-5 SR |  |  |  |
| Sex | SR |  |  | T1-5 SR |  |  |  |
| Marital status | SR |  |  | T1-5 SR |  |  |  |
| First language | SR |  |  | T1 SR |  |  |  |
| Study situation | SR |  |  | T4-5 SR |  |  |  |
| Height |  |  |  | T5 SR |  |  |  |
| Weight |  |  |  | T5 SR |  |  |  |
| Handedness |  |  |  | T5 SR |  |  |  |
| Sleeping habits |  |  |  | T5 SR |  |  |  |
| Sick days |  |  |  | T5 SR |  |  |  |
| Living situation |  |  |  | T3-5 SR |  |  |  |
| Favorite subjects |  |  |  | T5 SR |  |  |  |
| Romantic relationships |  |  |  | T5 SR |  |  |  |
| Going out frequencies |  |  |  | T5 SR |  |  |  |
| Life satisfaction |  |  |  | T5 SR |  |  |  |
| Staying abroad |  |  |  | T5 SR |  |  |  |
| Visited countries |  |  |  | T5 SR |  |  |  |
| Foreign languages |  |  |  | T5 SR |  |  |  |
| Smoking |  |  |  | T5 SR |  |  |  |
| Alcohol consumption |  |  |  | T5 SR |  |  |  |
| **Trait measures** |  |  |  |  |  |  |  |
| Big Five |  |  |  |  |  |  |  |
| - Neuroticism | SR, IR |  |  | T1-5 SR, T1 IR |  |  |  |
| - Extraversion | SR, IR |  |  | T1-5 SR, T1 IR |  |  |  |
| - Openness | SR, IR |  |  | T1-5 SR, T1 IR |  |  |  |
| - Conscientiousness | SR, IR |  |  | T1-5 SR, T1 IR |  |  |  |
| - Agreeableness | SR, IR |  |  | T1-5 SR, T1 IR |  |  |  |
| Shyness | SR, IR |  |  | T1-5 SR, T1 IR |  |  |  |
| Shyness toward other sex | SR, IR |  |  | T1-5 SR, T1 IR |  |  |  |
| Sociability | SR, IR |  |  | T1-5 SR, T1 IR |  |  |  |
| Extraversion toward other sex | SR, IR |  |  | T1-5 SR, T1 IR |  |  |  |
| Narcissism (NPI-40) | SR |  |  | T1-5 SR |  |  |  |
| Narcissism (NPI-15) | IR |  |  | T1 IR |  |  |  |
| Trait affect and self-esteem | SR, IR |  |  | T1-5 SR, T1 IR |  |  |  |
| Trait Anger | SR, IR |  |  | T1-5 SR, T1 IR |  |  |  |
| Impulsivity | SR, IR |  |  | T1-5 SR, T1 IR |  |  |  |
| Sensation seeking | SR, IR |  |  | T1-5 SR, T1 IR |  |  |  |
| NARQ: Narcissistic admiration | SR, IR |  |  | T1-5 SR, T1 IR |  |  |  |
| NARQ: Narcissistic rivalry | SR, IR |  |  | T1-5 SR, T1 IR |  |  |  |
| Dirty Dozen: Narcissism | SR, IR |  |  | T1-5 SR, T1 IR |  |  |  |
| Dirty Dozen: Machiavellianism | SR, IR |  |  | T1-5 SR, T1 IR |  |  |  |
| Dirty Dozen: Psychopathy | SR, IR |  |  | T1-5 SR, T1 IR |  |  |  |
| Self-concept |  |  |  |  |  |  |  |
| - Intellectual ability | SR, IR |  |  | T1-5 SR, T1 IR |  |  |  |
| - Social skills | SR, IR |  |  | T1-5 SR, T1 IR |  |  |  |
| - Artistic/ musical ability | SR, IR |  |  | T1-5 SR, T1 IR |  |  |  |
| - Athletic ability | SR, IR |  |  | T1-5 SR, T1 IR |  |  |  |
| - Leadership ability | SR, IR |  |  | T1-5 SR, T1 IR |  |  |  |
| - Common sense | SR, IR |  |  | T1-5 SR, T1 IR |  |  |  |
| - Emotional stability | SR, IR |  |  | T1-5 SR, T1 IR |  |  |  |
| - Sense of humor | SR, IR |  |  | T1-5 SR, T1 IR |  |  |  |
| - Discipline | SR, IR |  |  | T1-5 SR, T1 IR |  |  |  |
| - Attractiveness | SR, IR |  |  | T1-5 SR, T1 IR |  |  |  |
| - Specific intellectual abilities | SR, IR |  |  | T1-5 SR, T1 IR |  |  |  |
| - Agentic traits | SR, IR |  |  | T1-5 SR, T1 IR |  |  |  |
| - Communal traits | SR, IR |  |  | T1-5 SR, T1 IR |  |  |  |
| - Antagonistic Traits |  |  |  | T1-5 SR, T1 IR |  |  |  |
| Communal narcissism | SR, IR |  |  | T1-5 SR, T1 IR |  |  |  |
| Self-esteem | SR, IR |  |  | T1-5 SR, T1 IR |  |  |  |
| Need to belong | SR, IR |  |  | T1-5 SR, T1 IR |  |  |  |
| Sociosexual orientations | SR, IR |  |  | T1-5 SR, T1 IR |  |  |  |
| Sexual orientation | SR, IR |  |  | T1-5 SR, T1 IR |  |  |  |
| Implicit personality |  |  |  |  |  |  | SR |
| Social value orientation |  |  |  |  |  |  | SR |
| Cognitive abilities |  |  |  |  |  |  |  |
| - Working memory |  | S1 SR |  |  |  |  | SR |
| - Vocabulary knowledge |  | S1 SR |  |  |  |  | SR |
| - Reasoning |  | S1 SR |  |  |  |  | SR |
| Public Goods Game |  |  |  |  |  |  | SR |
| **Relationship indicators** |  |  |  |  |  |  |  |
| Acquaintance |  | S1 IP | IP |  | A IP |  |  |
| Friendship |  | S1-3 SR, IP |  | T3-T5 | C IP |  |  |
| Leadership |  | S1-3 SR, IP |  |  | C IP |  |  |
| Dating and mating potential |  | S3 SR, IP |  |  | C IP |  |  |
| Interaction frequency |  |  |  | T3-5 SR | B-C IP |  |  |
| Relationship quality |  |  |  | T2-T5 SR | C IP |  |  |
| Broader social network |  |  |  | T1-5 SR | A IP |  |  |
| Social interaction ratings |  |  |  | T3-5 SR |  | SR IP |  |
| **Interpersonal perceptions** |  |  |  |  |  |  |  |
| Liking |  | S1-3 SR, IP | IP |  | A SR, IP |  |  |
| Metaliking |  | S1-3 SR, IP | IP | T3-5 SR | A SR, IP |  |  |
| Annoying |  | S1-3 SR, IP |  |  |  |  |  |
| Attractiveness |  | S1-3 SR, IP |  |  |  |  |  |
| Personality impressions |  | S1-3 SR, IP | IP |  | B SR, IP |  |  |
| Status perceptions |  |  |  | T4-5 SR IP | C SR IP |  |  |
| Retrospective accuracy perceptions | | T3 SR |  | T3-5 SR |  |  |  |
| **Personality state ratings** |  |  |  |  |  |  |  |
| State affect |  | S1-3 SR | SR |  |  | SR |  |
| Affect grid |  | S1-3 SR |  |  |  |  |  |
| State self-esteem |  | S1-3 SR | SR |  |  | SR |  |
| **Physical and behavioral measures** | |  |  |  |  |  |  |
| Physical ratings |  |  |  |  |  |  |  |
| - Face |  | OR | OR |  |  |  | OR |
| - Body |  | OR | OR |  |  |  | OR |
| - Clothes |  | OR | OR |  |  |  | OR |
| - Hair |  | OR | OR |  |  |  | OR |
| Individual behavioral ratings |  |  |  |  |  |  |  |
| - Attention (Voice) |  | S1 OR |  |  |  |  |  |
| - Cheerfulness of voice |  | S1 OR |  |  |  |  |  |
| - Intelligence (Voice) |  | S1 OR |  |  |  |  |  |
| - Verbal fluency (Voice) |  | S1 OR |  |  |  |  |  |
| - Nervousness (Voice) |  | S1 OR |  |  |  |  |  |
| - Arrogant behavior |  | S1-3 OR | OR |  |  |  | OR |
| - Dominant behavior |  | S1-3 OR | OR, IP |  |  |  |  |
| - Expressive behavior |  | S1-3 OR | OR |  |  |  | OR |
| - Friendly behavior |  | S1 OR | OR |  |  |  |  |
| - Nervous behavior |  | S1 OR | OR |  |  |  | OR |
| - Aggressive behavior |  | S2-3 OR |  |  |  |  |  |
| - Cooperative behavior |  | S2-3 OR |  |  |  |  |  |
| - Warm-heartedness |  |  |  |  |  |  | OR |
| - Self-confidence |  |  |  |  |  |  | OR |
| - Intellectual behavior |  |  |  |  |  |  | OR |
| - Affectionate behavior |  |  | IP |  |  |  |  |
| Group behavioral ratings |  |  |  |  |  |  |  |
| - Performance |  | S2-3 OR |  |  |  |  |  |
| - Frequency of conflicts |  | S2-3 OR |  |  |  |  |  |
| - Positive atmosphere |  | S2-3 OR |  |  |  |  |  |
| Interactional behavioral ratings |  |  |  |  |  |  |  |
| - Dominant vs. submissive | |  |  |  |  | SR, IP |  |
| - Sociable vs. reclusive | |  |  |  |  | SR, IP |  |
| - Friendly vs. unfriendly | |  |  |  |  | SR, IP |  |
| - Arrogant vs. modest | |  |  |  |  | SR, IP |  |
| - Exploiting vs. cooperative | |  |  |  |  | SR, IP |  |
| - Self-revealing vs. reserved | |  |  |  |  | SR, IP |  |
| - Reliable vs. unreliable | |  |  |  |  | SR, IP |  |
| **Additional life events & Outcomes** | |  |  |  |  |  |  |
| Social network use | SR |  |  | T1-5 SR |  |  |  |
| Academic achievements |  |  |  | T3-5 SR |  |  |  |
| Life events |  |  |  |  |  |  |  |
| - Family |  |  |  | T5 SR |  |  |  |
| - Friends |  |  |  | T5 SR |  |  |  |
| - Romantic relationships |  |  |  | T5 SR |  |  |  |
| - Personal life |  |  |  | T5 SR |  |  |  |
| - Work |  |  |  | T5 SR |  |  |  |
| - Studies |  |  |  | T5 SR |  |  |  |
| Self-perceived personality development | |  |  | T3-5 SR |  |  |  |

*Note. Zero Acqu. =* zero-acquaintance experiment. SR = self-report, IR = informant-report, IP = interpersonal perception, OR = observer rating, S = sessions 1 to 3, T = time points 1 to 5.
