# Supplementary material for: Explaining the longitudinal interplay of personality and social relationships in the laboratory and in the field: The PILS and the CONNECT study
Source: PLoS One. 2019 Jan 30;14(1):e0210424. doi: 10.1371/journal.pone.0210424 (PMC6353144; doi:10.1371/journal.pone.0210424)
Supplement: S3 Table — (DOCX) [file pone.0210424.s003.docx]

**Supporting Information 3**

**Table S3. Overview of assessed life events in CONNECT**

| Family | Family member got seriously ill or badly injured | | |
| --- | --- | --- | --- |
|  | Family member passed away | | |
|  | Parents split up or got divorced | | |
|  | Closeness to family members changed | | |
|  | Family gained a new family member | | |
| Friends | Close friend got seriously ill or badly injured | |  |
|  | Close friend passed away | | |
|  | Made a new close friend | | |
|  | Serious conflict with a close friend | | |
|  | Gave up a close friendship | | |
| Romantic Relationships | Started being in a relationship |  |  |
|  | Partner got badly injured or was seriously ill | | |
|  | Partner passed away | | |
|  | Serious relationship crisis | | |
|  | Number of conflicts with partner changed | | |
|  | Problems with your partner’s family | | |
|  | Partner problems with participant’s family | | |
|  | Romantic relationship to former partner ended | | |
|  | Made up after a serious relationship crisis | | |
|  | Engaged | | |
|  | Married | | |
|  | Divorced | | |
|  | Spatial proximity or distance to partner changed | | |
|  | Partner’s work habits significantly changed | | |
|  | Participants’/partners’ pregnancy | | |
|  | Participants’/partners’ abortion | | |
|  | Sexual problems | | |
| Personal Life | Got badly injured or was seriously ill | | |
|  | Personal meaningful events | | |
|  | Spent at least 1 month at a stretch abroad for no professional or education-related reasons (private) | | |
|  | Spent at least 1 month abroad, for example, due to an internship or your studies | | |
|  | Time spend on social activities (e.g., parties, cinema, cultural activities, cooking together) changed | | |
|  | Type of social activities changed | | |
|  | Changed sleeping habits | | |
|  | Changed quantitative eating habits | | |
|  | Changed qualitative eating habits | | |
|  | Changed alcohol consumption habits | | |
|  | Changed smoking habits | | |
|  | Moved out from parental home | | |
|  | Moved (apart from moving out from parental home) | | |
|  | If lived in a shared flat: serious problems in shared flat | | |
|  | Financial situation significantly changed | | |
|  | Problems with living expenses or concerned about not being able to finance the continuation of studies | | |
|  | Borrowed a major amount of money (more than 1,000 Euro) from someone (apart from BAfÖG or something similar) | | |
|  | Went through a compulsory execution or pledging of goods | | |
|  | Got caught because of a minor breaking of law (e.g., fare dodging, speeding, etc.) | | |
|  | Been in jail or a comparable institution (e.g., a drunk tank, a psychiatric clinic, or something similar) | | |
|  | Went to or started psychotherapy | | |
|  | Failed an important nonuniversity exam (e.g., driving test) | | |
|  | Important nonuniversity exam (e.g., driving test) not attended | | |
| Work | Worked before starting studies or worked part-time while studying or accepted a new job | | |
|  | Working hours at job increased significantly | | |
|  | Promoted or responsibility at job significantly increased | | |
|  | Conflicts with employer which brought up the risk of losing job, being suspended or being demoted to a lower rank | | |
|  | Quit job (by participant or employer) | | |
| Studies | Dropped out or changed course of studies or apprenticeship | | |
|  | Graduated from high school | | |
|  | Failed an university exam | | |
|  | Did not attend an important university exam | | |
|  | Doubts about continuing studies | | |
|  | Transferred to another university | | |
|  | Started another academic course (at university, technical college, or training school) above studies of psychology | | |
|  | Graduated from an academic course other than psychology course | | |
| Other | Any further incident that had an impact on life | | |
